# Liquid Biopsy Analysis of the EV-Associated Micro-RNA Signature in Vulvar Carcinoma May Benefit Disease Diagnosis and Prognosis

**DOI:** 10.3390/cancers18030438

**Published:** 2026-01-29

**Authors:** Friederike Borchardt, Leonie Kleinholz, Anna Jaeger, Jana Löptien, Vanessa Vohl, Jolanthe Kropidlowski, Klaus Pantel, Eik Vettorazzi, Linn Woelber, Harriet Wikman, Katharina Effenberger

**Affiliations:** 1 Department of Tumor Biology, University Medical Center Hamburg-Eppendorf, Martinistr. 52, 20246 Hamburg, Germany; friederike.borchardt@stud.uke.uni-hamburg.de (F.B.); leonie.kleinholz@stud.uke.uni-hamburg.de (L.K.); j.loeptien@uke.de (J.L.); vanessa.vohl@stud.uke.uni-hamburg.de (V.V.); j.kropidlowski@uke.de (J.K.); pantel@uke.de (K.P.); h.wikman@uke.de (H.W.); 2 Department of Gynecology, University Medical Center Hamburg-Eppendorf, 20246 Hamburg, Germany; a.jaeger@uke.de (A.J.); l.woelber@uke.de (L.W.); 3 Department of Medical Biometry and Epidemiology, University Medical Center Hamburg-Eppendorf, 20246 Hamburg, Germany; e.vettorazzi@uke.de

**Keywords:** vulvar cancer, liquid biopsy, extracellular vesicles (EV), micro-RNA (miRNA), EV-associated micro-RNA, EV-enriched miRNA fractions, exosomal micro-RNA, vulvar carcinoma, tumor biomarker, HPV

## Abstract

**Background:** Vulvar cancer mainly affects postmenopausal women, but its incidence is rising among younger individuals due to persistent HPV infection. Validated diagnostic biomarkers remain lacking, though circulating exosomal microRNAs (exomiRs) have recently emerged as promising liquid biopsy tools across various cancers. **Objective:** The purpose of this study was to identify a panel of dysregulated plasma-derived extracellular vesicle (EV)-associated miRNAs, hereafter referred to as exosomal micro-RNAs, as liquid biopsy markers for the detection of vulvar cancer and for assessment of HPV-positivity. **Methods:** Five healthy donor (HD) and 10 vulvar cancer samples underwent Next-Generation Sequencing to screen for differentially expressed exomiRs. The seven most dysregulated and four stably expressed exomiRs were subsequently analyzed in 81 cancer and 60 HD samples by qRT-PCR. Differential expression was determined by the 2^−ΔΔCT^ method. Binary regression was used to construct an exomiR panel. HPV status was assessed using mass spectrometry. **Results:** Five single exomiRs showed a statistically significant dysregulation in cancer patients compared to healthy controls: miR-143-3p, miR-223-3p, miR-451a, miR-4516 and miR-151a-5p. The combination of six exomiRs resulted in a panel with superior diagnostic ability (*p* < 0.001; ROC-AUC = 0.805; 95% CI: 0.726–0.884) in distinguishing cancer patients from HDs. A model consisting of miR-223-3p, miR-143-3p and miR-451a could discriminate HPV-positive from -negative (*p* = 0.003; ROC-AUC = 0.939), and a model of miR-4516, miR-143-3p, miR-16-5p and miR-451a was predictive of lymph node positivity (*p* < 0.001, ROC-AUC = 0.786). Multivariate Cox regression showed that a model of downregulated miR-16-5p and upregulated miR-451a was significantly associated with poorer survival (*p* = 0.023). **Conclusions:** This study indicates the future potential of exomiRs as diagnostic and prognostic liquid biopsy markers for vulvar cancer.

## Simple Summary

Vulvar cancer is a rare gynecological tumor, but recently it has become more common in younger people, mainly due to persistent human papillomavirus (HPV) infection. Research is limited and, to date, there are neither standardized screening tools nor diagnostic biomarkers available. Therefore, the aim of this study was to detect dysregulated extracellular vesicle–associated miRNAs, hereafter referred to as exosomal micro-RNAs (exomiRs) as a blood-based liquid biopsy marker for vulvar cancer. Using modern laboratory techniques, it was possible to identify a set of specific exomiRs which could distinguish cancer patients from healthy volunteers. Additionally, certain exomiRs were found to be associated with HPV positivity, lymph node metastasis and the presence of precursor lesions, which could be useful for risk stratification of patients. This study shows the future potential of exomiRs in the assessment of vulvar cancer and is a first step towards personalized approaches in the clinical assessment of this often-neglected disease.

## 1. Introduction

### 1.1. Vulvar Cancer

Vulvar cancer is a rare genital tumor which arises from the outer female genitalia affecting mainly postmenopausal women. With an annual incidence of 2.5–4.4/100,000 in developed Western countries, it ranks fourth of all gynecological malignancies [1]. However, in several high-income countries, an increasing incidence—especially among women under 60—has been observed over recent decades, likely driven by persistent high-risk human papillomavirus (HPV) infections [2,3,4]. Vulvar squamous cell carcinoma (VSCC) is the most common histological subtype, which in most cases evolves from squamous non-invasive precursor lesions called vulvar intraepithelial neoplasia (VIN) [5,6,7]. Vulvar cancer may present with no or only unspecific symptoms, such as erythematous or ulcerous lesions, pruritus, pain, bleeding, dysuria or vaginal discharge [8,9].

Next to HPV infection, the most important risk factors for the development of VSCC include age, smoking, chronic dermatoses and atrophic conditions like lichen sclerosus and immunodeficiency [3,5,8]. There are two distinguished types of VSCC: HPV-dependent VSCC and HPV-independent VSCC, mainly based on TP53 mutations as the oncogenic driver [6]. Vulvar cancer is staged into four stages, pursuant to revised FIGO (International Federation of Gynecology and Obstetrics) classification from 2021. Radical surgery remains the primary curative therapy option, since no tumor-tailored treatment options exist [1,8,10].

The main prognostic factors for overall survival comprise tumor stage, nodal status, tumor size and depth of invasion. Adjuvant radio- or chemotherapy are beneficial in cases with positive lymph nodes [1,11].

In 2023, the European Society of Gynecological Oncology presented an update on their guidelines for the management of vulvar cancer patients, which reaffirmed that the diagnosis relies on clinical examination and is confirmed by tissue biopsy [12]. There are no established screening or prevention programs or a specific non-invasive biomarker, neither for vulvar cancer nor for its precursor lesions.

Few studies have investigated the utilization of blood-based biomarkers as potential diagnostic or prognostic tools for vulvar cancer. One study found that serum concentrations of squamous cell carcinoma antigen (SCC-Ag) may be an additional independent prognostic factor for survival [13,14]. Another study stated that SCC-Ag and carcinoembryonic antigen (CEA) are useful to predict lymph node metastasis and recurrence, and that they reflected the response to neoadjuvant chemotherapy [15]. In contrast, two studies also evaluated SCC-Ag levels in VSCC patients and found no preoperative correlation of SCC-Ag levels and lymph node metastasis and that its determination does not provide additional information in staging [16,17]. The contradictory findings may explain why none of the biomarkers analyzed were transferred into clinical practice.

### 1.2. Extracellular Vesicle-Associated Micro-RNAs as Liquid Biopsy Markers in Vulvar Cancer

The general concept of liquid biopsy in oncology is the detection and analysis of tumor-derived material obtained out of the systemic circulation which allows deduction about its origin and provides information about the cancer, while mitigating the risk of invasive tissue biopsy.

In recent years, micro-RNAs (miRNAs) gained increasing attention for their potential as tumor-specific liquid biopsy markers. MiRNAs are small non-coding RNA molecules, comprising 18–22 nucleotides, which play a significant role in post-transcriptional gene regulation and are involved in various physiological and pathophysiological processes [18]. MiRNAs are released by their donor cell into systemic circulation either as cell-free molecules or inside of membrane-coated extravesicular vesicles (EVs) that protect their cargo from enzymatic degradation. The best-known and predominantly studied subfraction of EVs are exosomes, with a diameter of 30 to 150 nm. Their lipid bilayer membrane increases their stability in circulation and enables regulated secretion and targeted uptake of their cargo by recipient cells, thereby facilitating controlled intercellular communication. Hence, exosomal-encapsulated miRNAs (exomiRs) are involved in cell-to-cell crosstalk and are part of multiple molecular signaling networks.

They play a pivotal role in tumor biology by modulating key processes and signaling pathways involved in cancer progression, metastasis or resistance mechanisms. Moreover, they contribute to tumorigenesis by influencing the tumor microenvironment, priming of metastatic niches and promoting processes like angiogenesis [19,20,21,22].

ExomiR expression is often altered in the blood of cancer patients. It is known that diverse tumor entities express unique and specific patterns of exomiR which are associated with stage, grade, prognosis or other clinicopathologic characteristics, making them valuable as highly reliable liquid biopsy markers for the diagnosis and monitoring of malignant processes [18,23,24,25,26]. New technologies are undergoing rapid development [21,26]. For several gynecological cancers, their diagnostic utility has already been acknowledged [27,28,29,30]. For example, exomiRs were found dysregulated in ovarian, cervical and endometrial carcinoma and were proposed to be useful as accurate diagnostic and prognostic biomarkers, especially powerful if several of them are combined in a panel [28,30,31,32]. However, research about circulating biomarkers in vulvar cancer is very limited. Two sequential studies [33,34] focused on cell-free miRNAs (cf-miRNAs) in plasma of VSCC and VIN patients as well as non-affected control persons, and found that miR-431-5p increased in the plasma of VSCC compared to VIN patients. Also, they reported that a low level of circulating miR-431-5p was predictive for poor survival rates. The second study identified, in total, 31 differentially expressed cf-miRNAs when comparing VSCC to patients with premalignant lesions.

However, preceding evidence from other cancer entities suggests that exomiRs hold several advantages as tumor biomarkers compared to other analytes as cf-miRNA, CTCs or circulating tumor DNA. Due to their highly regulated and selective form of enrichment and liberation, exomiRs might be more specific for the underlying tumor entity, reflecting its molecular makeover [35,36,37,38,39].

To the best of our knowledge, the role of exomiRs in vulvar cancer has not been characterized yet. Due to missing biomarkers and the lack of established screening programs, there is a delay in diagnosis, leading to a poorer outcome for affected patients. Also, precancerous lesions and risk factors such as lichen sclerosus are often missed out and not adequately treated, which contributes to a rising incidence especially in younger women [2,3,4].

As mentioned above, the genital infection with high-risk HPV types is associated with the development of VSCC. There is no exact data for rates of HPV-positivity in vulvar cancer, but estimations range from 39% to 69% [4,8,40,41]. HPV-associated gene dysregulation through E6/E7 affects miRNA expression and causes aberrations in the concentration of intracellular as well as circulating miRNAs [42,43,44,45,46,47]. It is known that miRNA expression in VSCC tissue differs depending on HPV status of the tumor [48,49], but, to date, there are no published studies that investigated HPV-driven changes of exomiR expression in HPV-positive VSCC.

Taken together, no liquid biopsy markers for vulvar cancer or its precursor lesions have been identified yet. The adoption of emerging liquid biopsy strategies could help to improve the assessment and work-up of this marginalized disease. Therefore, the aim of this experimental two-phase-study was to first identify the most dysregulated EV-associated miRNAs in plasma of vulvar cancer patients and secondly test them as single markers as well as combined in a panel for their ability to serve as diagnostic biomarkers.

## 2. Materials and Methods

### 2.1. Study Design

This experimental study consisted of two phases: A screening and a validation phase. First, to find out differentially expressed exomiRs in vulvar cancer patients compared with non-affected females, plasma samples of 11 vulvar cancer patients and of 5 female healthy donors (HDs) were sent out for Next-Generation Sequencing (NGS), which was executed externally at Qiagen (Hilden, Germany). The target and housekeeping candidates were selected as targets for further analysis in the validation cohort. In the second phase, the exomiRs of 81 vulvar cancer patients and 60 female HDs were isolated and quantified using a customized polymerase chain reaction (PCR) panel designed by Qiagen. The data were normalized to the most stable internal control with the 2^−∆∆Ct^ method and statistically evaluated to build a diagnostic exomiR panel.

Additionally, tumor DNA was isolated from formalin-fixed paraffin-embedded (FFPE) material of 44 VSCC patients, and HPV subtypes were determined using the mass spectrometry-based MassArray typer (Agena Bioscience, Hamburg, Germany).

### 2.2. Sample Size Analysis

Prior to the enrolment of patients, a sample size analysis was conducted to calculate an appropriate cohort size achieving enough power for statistical analysis. The statistical power was set at 80%, with a significance level of α = 0.05. Considering the published literature about similar trials, a mean difference of raw Cq-values of approximately one between healthy controls and vulvar carcinoma patients was estimated [50,51]. The standard deviation was set at 1.1 for the healthy control cohort and at 1.6 for the cancer patient cohort. A *t*-test was performed, which resulted in a required cohort size of 72 for vulvar cancer and at least 22 for HD samples with *p* < 0.05. To ensure the likelihood of statistical power with regard to possible dropouts, and to provide a balanced relation of vulvar cancer and HD samples on each PCR panel, a final number of 81 cancer samples versus 60 HD samples was included.

### 2.3. Patient Recruitment and Blood Sample Collection

This study included a total of 141 plasma samples, of which 81 samples originated from cancer patients, who were treated at the Department of Gynecology, University Medical Centre Hamburg-Eppendorf. Inclusion criteria for cancer patients were informed written consent by the patient, confirmed diagnosis of vulvar carcinoma, at least 5 mL of EDTA blood, and availability of clinical data. Exclusion criteria were insufficient amount of biomaterial, different diagnosis being confirmed, second primary malignancy or lack of clinical data. The 60 control samples were donated from healthy females who were recruited during routine blood donation at the Department of Transfusion Medicine, University Medical Centre Hamburg-Eppendorf. The hereafter used acronym HD (healthy donor) refers to female volunteer donors without known underlying conditions, meeting health eligibility criteria for blood donation. The realization of the project was pre-approved by the ethics committee (Ethik-Kommission der Ärztekammer, Hamburg, Germany, PV5392). Vulvar cancer patients were recruited between September 2016 and June 2023, and 2023 and gave their written informed consent. All procedures were performed in compliance with the Declaration of Helsinki.

Blood samples of vulvar cancer patients were acquired directly prior to the primary surgical intervention in 72 cases (88.9%), and in 9 cases, blood was drawn during their gynecological visit or before the start of adjuvant radiochemotherapy. All cancer diagnoses were histopathologically confirmed by tissue biopsy. In synopsis of their clinical diagnostic findings, imaging and biopsy, the patients were assigned to a FIGO stage. Vulvar cancer patients were followed up until May 2024, and only events in this time period were included in survival analysis. The date is hence censored for this time point. The follow-up data of 76 patients was available and included in the analysis of endpoints. Based on the acquired data, the patients were assigned to one of the categories that were determined as end-point-events: death (*n* = 22, 28.9%); recurrence or disease progression (*n* = 5, 6.6%) or non-disease-related-event-survival (date of last follow-up request) (*n* = 49, 64.5%).

Blood samples were collected in 7.5 mL EDTA tubes and were transferred to the laboratory, undergoing a double blinding for anonymization. Afterwards, they were processed within two hours under sterile conditions. Plasma was isolated by double centrifugation at 300× *g* and 1800× *g* for 10 min each, and it was checked visually for hemolysis before storing at −80 °C until further utilization

### 2.4. Next-Generation Sequencing and Selection of Target exomiRs

As stated above, 11 vulvar cancer plasma samples and 5 HD plasma samples were randomly selected and sent to Qiagen (Hilden, Germany), who performed Next-Generation Sequencing (NGS). In short, RNA was isolated from 1000 µL using the exoRNeasy Maxi (Qiagen) according to manufacturer’s instructions. The library preparation was done using the QIAseq miRNA Library Kit (Qiagen). A total of 5 μL RNA was converted into miRNA NGS libraries. After adapter ligation, UMIs were introduced in the reverse transcription step. The cDNA was amplified using PCR (22 cycles), and during the PCR, indices were added. After PCR the samples were purified. Library preparation was quality controlled using capillary electrophoresis (Tape D1000). Based on the quality of the inserts and the concentration measurements, the libraries were pooled in equimolar ratios. The library pools were quantified using qPCR and then sequenced on a NextSeq (Illumina Inc., San Diego, CA, USA) sequencing instrument according to the manufacturer instructions (1 × 75, 2 × 10). Raw data was de-multiplexed, and FASTQ files for each sample were generated using bcl2fastq2 software (Illumina Inc.), followed by reverse transcription, cDNA amplification, purification and quality-controlled library preparation. Primary analysis was carried out using CLC Genomics Server 21.0.4. The workflow “QIAseq miRNA Quantification” of CLC Genomics Server with standard parameters was used to map the reads to miRBase version 22. The percentage of reads that were successfully mapped ranged from 26.05–77.01%. The number of mapped reads was sufficient for all downstream analysis. All reads which did not map to miRBase were mapped to the human genome (hg38). This was carried out using the “RNA-Seq Analysis” workflow of CLC Genomics Server with standard parameters. The mapping rate was between 67.27–77.11%, with the largest proportion of these reads mapping to protein coding genes and lncRNAs.

The “Empirical analysis of DGE” algorithm of the CLC Genomics Workbench 21.0.4 was used for differential expression analysis with default settings. It is an implementation of the “Exact Test” for two-group comparisons and incorporated in the EdgeR Bioconductor package.

For all unsupervised analysis, only miRNAs were considered, with at least 10 counts summed over all samples. A variance stabilizing transformation was performed on the raw count matrix using the function vst of the R package DESeq2 version 1.28.1. A total of 500 genes with the highest variance were used for the principal component analysis. The variance was calculated agnostically to the predefined groups (blind = TRUE). A total of 35 genes with the highest variance across samples were selected for hierarchical clustering.

One cancer sample was excluded from the analysis due to hemolysis, so expression analysis was performed on 10 cancer patients versus 5 HDs. Differential expression analysis results between vulvar cancer and HD samples were provided by Qiagen. The top 7 most dysregulated exomiRs in cancer patients were selected as targets for further analysis in the validation cohort. Additionally, 4 of the most stably expressed exomiRs among both cohorts were selected as potential internal control genes.

The selected exomiRs were subjected to a preliminary PubMed database screening for previous reports in tumor biomarker studies, especially with regards to exomiR quantification as diagnostic liquid biopsy tool using qPCR and with regards to studies which investigated the functional role of the exomiRs. All target exomiRs have already been reported in the context of malignant diseases, except for miR-12135. This mature exomiR is annotated in miRbase (The miRBase Sequence Database—Release 22.1) as only experimentally confirmed. A brief synopsis of this literature research is outlined in the Supplementary Materials (Supplementary File S1). References [52,53,54,55,56,57,58,59,60,61,62,63,64,65,66,67,68,69,70,71] are cited in the Supplementary Materials S1.

A customized qPCR panel containing preformed primers was designed, comprising target and internal control exomiRs (miRCURY Custom PCR Panel 96-well plates, Qiagen, cat. No. 339332). The panel was tested with plasma samples of 81 vulvar cancer patients and 60 HDs.

The selected target exomiRs were: miR-4516 (GeneGlobe ID: YP02112882), miR-16-5p (YP00205702), miR-143-3p (YP00205992), miR-451a (YP02119305), miR-151a-5p (YP00204007), miR-223-3p (YP00205986) and miR-12135 (YP02126972) (details in Supplementary File S3).

### 2.5. RNA Isolation and cDNA Synthesis

Prior to the RNA isolation, plasma was pre-filtered using a Millipore Millex AA Syringe filter (Merck Millipore, Burlington, MA, USA), with a pore size of 0.8 µm, according to the manufacturer’s recommendation, and a diameter ø of 33 mm to eliminate larger particles and platelets [72,73,74].

The purification of the EV-related RNA was performed using the exoRNeasy Midi Kit (Qiagen cat. No.77144, Hilden, Germany). The starting plasma volume was 800 µL. The RNA was eluted in 140 µL of RNAse-free water and stored at −80 °C until further utilization. The complimentary DNA (cDNA) synthesis was performed using the miRCURY LNA RT-kit (Qiagen, cat. No. 339340, Hilden, Germany) with an initial template RNA volume of 1.4 µL, which was calculated using the recommended estimation of Qiagen (Supplementary File S2). All protocols were executed according to the manufacturer’s instructions. The cDNA was stored at −15 °C to −30 °C for a maximum of 5 weeks until further utilization.

A detailed description of the workflow is provided in the Supplementary Materials (Supplementary File S2). Reference [75] is cited in the Supplementary File S2. For detailed information regarding the components of the reagents, please consult the manufacturer’s handbooks and product specifications (miRCURY LNA miRNA SYBR^®^ Green PCR Biofluid Samples Handbook 10/2019, HB-2439-002).

### 2.6. qPCR Quality Control

Before running the qPCR Custom panels, each sample was subjected to a quality control using the miRCURY LNA miRNA QC PCR Panel (cat. no. 339331). Each panel comprised 96 wells with 12 different assays configured for 8 individual samples. The process protocol for the QC panel was the same as for the customized panel, with a total input amount of 2.5 µL of diluted cDNA per sample, which corresponded to a volume of 0.2 µL of cDNA per assay. The QC panel served to observe the process performance and technical execution of the RNA purification as well as to assess the biological quality of the samples. A comprehensive listing of its included items can be found in the respective handbook (miRCURY LNA miRNA QC PCR Panel Handbook 10/2017, HB-2440-001). All samples passed this quality control and were subsequently admitted to the following analysis with the customized panel.

The kits used for exosome purification and RNA isolation are commercially supplied by Qiagen and proven by multiple preceding studies to deliver robust and reliable results [39,76,77,78,79,80,81]. The technical success of RNA isolation was additionally confirmed by Spike-ins and the QC Panel (Supplementary File S2).

The cycler used for all PCR reactions was BioRad CFX, performing dye-based PCR with SYBR Green and using the 2× miRCURY SYBR Green master mix. The cycler algorithm was programmed the following way: The polymerase initial heat activation took place at 95 °C for 2 min, followed by a 2-step cycle sequence which consisted of the denaturation at 95 °C for 10 s and the combined annealing and extension phase at 56 °C for 60 s. Both steps were repeated for a total of 40 cycles. Data acquisition was performed at the end of every cycle.

### 2.7. Custom Panel

Quantitative real-time PCR was performed using the miRCURY LNA miRNA Custom PCR Panel (cat. nos. 339330, 339332, Qiagen, Hilden, Germany) and the miRCURY LNA SYBR Green PCR Kit (cat. no. 339347). Each 96-well panel was configured for 8 samples and 12 assays per sample. For each sample, it contained primers for the 7 selected target exomiRs and 4 of the selected internal controls for later normalization. Additionally, for each sample, one well was reserved for an interplate calibrator. For each well, a template volume of 0.625 µL of cDNA was used. This volume was determined after conducting several test runs to obtain Cq-values in an optimal range between 20 and 30. For the miRCURY Custom panel, UniSP3 was used as interplate calibrator to monitor PCR efficiency. The cycler algorithm was programmed the same way as for the QC protocol (see description above).

For one target exomiR (miR-12135) that was not detected on the panel, an exception was made by employing an RT-PCR single assay with increased input volumes (for details please see the description in the Supplementary File S2).

### 2.8. Selection of Internal Controls for Data Normalization

Next to the identification of dysregulated exomiRs, NGS was also used to detect stably expressed exomiRs, which could serve as potential internal controls for normalization of qPCR data. Four candidates were selected based on their lowest counts and standard deviation using NormFinder for miR-378a-3p, miR-361-5p, miR-181-5p and miR-30e-5p (the latter based on Qiagen NGS recommendations). They were then quantified together with the target genes using the customized qRT-PCR panels (Qiagen, Hilden, Germany). With a mean delta Cq-value of only 0.14 and an overall standard deviation of 1.43, miR-378a-3p showed the lowest delta and the lowest SD of calibrated Cq-values when comparing HD mean values with vulvar cancer mean values. Therefore, miR-378a-3p was selected as the best internal control for subsequent normalization.

Although miR-378a-3p is known to be differentially regulated in other pathophysiological contexts and a functional implication in some tumor entities cannot be ruled out, it was strictly used for normalization in this study. Its eligibility as endogenous control is supported by stable expression in both NGS and PCR analysis. However, it should be kept in mind that it could be confounded in patients suffering from more than one disease at the same time.

### 2.9. DNA Isolation from FFPE Tissue

Tumor samples were acquired intraoperatively, and FFPE samples were stored postoperatively at the Department of Pathology, University Hospital Hamburg-Eppendorf in case of all patients who underwent surgery at the UKE (*n* = 72). Later, 44 of the tumor samples could be retrieved and were provided for MassArray HPV analysis. Each sample was visually checked for the presence of tumorous parts. The FFPE tissue was then deparaffinized using a series of descending concentrations of ethanol (for detailed protocol please see Supplementary File S2). DNA was extracted using the black PREP FFPE DNA Kit (Analytik Jena GmbH & Co. KG, Jena, Germany). The procedure was performed following the manufacturer’s instructions (Publication No.: HB_BP-0021_e_180808 1). The concentration of eluted DNA in each eluate was determined using Qubit 4 and the Invitrogen Qubit dsDNA HS-Assay (Thermo Fisher Scientific, Waltham, MA, USA). The purified DNA was stored at −20 °C until further use.

### 2.10. HPV Genotyping

The HPV measurement was conducted using the Agena Bioscience HPV Genotyping Panel v.2.0 on the MassARRAY^®^ System (MassArray Analyzer 4, Agena Bioscience, San Diego, CA, USA), which is a matrix-assisted laser desorption ionization time-of-flight (MALDI-TOF) mass spectrometer. The panel is a single well assay, which can detect 24 different HPV subtypes in one multiplex assay, including 12 high-risk, carcinogenic subtypes, 1 probably carcinogenic, 7 possibly carcinogenic and 4 low-risk, not carcinogenic subtypes, as well as an internal control (GAPDH), to confirm the presence of DNA in the reaction. An overview of the included HPV types can be found in the manufacturer’s protocol. For the assay panel, premixed HPV PCR primers were used, which are based on type-specific sequences in the genomic E6/E7 region for 24 types. The panel also contained 4 negative controls of water and two positive controls of DNA from a cervical cancer cell line (Hela cell line), which is positive for HPV18.

The MassArray protocol comprises 3 steps, a PCR amplification, the shrimp–alkaline–phosphatase (SAP) reaction, and the iPLEX Pro Extension Reaction. Then, the mass spectrometry is performed and the MassArray launches an automated report from within the MassArray Typer software (Typer Analyzer 5.0.10.175), which provides all positive and negative HPV types. All reactions were set up and performed following the manufacturer’s instructions (iPLEX Pro Reagent Set), with minor adaptations to ensure adequate processing of samples even with low DNA concentrations. The protocol used has been validated before in cervical cancer [82]. For detailed information regarding the reaction cocktails and the protocol workflow, please see the tables and descriptions in the Supplementary Information (Supplementary File S2).

### 2.11. Statistical Analysis

Statistical analysis was performed with IBM SPSS Statistics version 29.0.1 for Windows (SPSS Inc, IBM Corp. Armonk, New York, NY, USA). The graphs of relative gene expression were generated using GraphPad Prism 10 (GraphPad Software, San Diego, CA, USA). The patient’s blood samples were acquired by the gynecological department and transferred to the lab blinded and anonymized. The patient data was also acquired by the clinicians of the gynecological department. It was then blinded and transferred to the lab, where it was analyzed anonymously. Missing clinical data was handled with pairwise deletion throughout all statistical analysis.

First, all raw Cq-values acquired by qPCR were subjected to interplate calibration, to eliminate the confounding effects of variations in qPCR efficacy. The fold change and fold change ratio were calculated using the 2^−∆∆Ct^ method, relative to the selected internal control miR-378a-3p (ΔCq = CqexomiR − Cqcontrol; ΔΔCq = ΔCqexomiR − ΔCqcontrol; Cq = the threshold cycle) [78].

There were three missing values for the endogenous control (miR-378a-3p), which were replaced with the respective mean Cq-value of the cohort. The missing Cq-values of the target exomiRs, in total 8 Cq-values, were excluded from statistics by pairwise deletion.

A symmetrical distribution of the log2 fold change values was assessed via visual examination of the histogram and the Q-Q-plots. A two-tailed *t*-test was applied to all datasets to compare the relative gene expression levels. To evaluate the diagnostic value of the exomiRs, Receiver Operating Characteristic (ROC) curve analyses were performed, Areas Under the Curve (AUCs) were generated, and the results are shown graphically. AUCs were calculated to assess their discriminatory ability between cancer patients and healthy controls. A higher AUC indicates better discrimination; however, no formal statistical test was applied to compare AUCs. Differences between AUCs are therefore reported descriptively. To report sensitivity and specificity, an optimal cut-off value was determined by maximizing the Youden index.

As our analyses have an exploratory character to generate hypotheses for future studies, we opted not to apply formal alpha-adjustment methods to avoid an excessive increase of type II errors and to not obscure potentially relevant findings [83]. Instead, we have transparently reported all *p*-values and effect estimates along with confidence intervals, thereby allowing to assess the strength and consistency of the results.

Binary logistic regression was used to create a panel of exomiRs with highest sensitivity and specificity. From the logistic regression model, a risk score can be derived, which was subsequently evaluated as a predictor in a ROC curve analysis. The predicted probabilities of the model were calculated, and a ROC analysis for the panel was conducted. AUCs were calculated with 95% confidence intervals (CI). A two-sided *p* < 0.05 was considered statistically significant

A bivariate correlation analysis was conducted to analyze the correlation between exomiR expression and age of the participants by applying Pearson’s correlation coefficient and a two-tailed *t*-test. The correlations were also checked visually and with a regression line in a scatterplot. Analysis of covariance was conducted to assess differential gene expression while considering age as potential confounding factor. Median follow-up time was estimated using the reverse Kaplan–Meier method, where censoring (alive at last follow-up) was treated as the event, and death was treated as a censored observation. The interquartile range (IQR) was calculated from the 25th and 75th percentiles. Kaplan–Meier analysis was performed to assess the median survival time of the patients. Multivariate Cox regression was used for survival analysis, with exomiR expression levels and lymph node status as covariates. The data is right censored for all events after the endpoint of the follow-up period.

The results are reported in accordance with the Guidelines for Statistical Reporting in Medical Journals [84]. The study was carried out in accordance with The Reporting Recommendations for Tumor Marker Prognostic Studies (REMARK) guidelines [85].

Artificial Intelligence (ChatGPT Version 5.2., Open AI) has been used to generate icons inserted in the graphical abstract.

## 3. Results

### 3.1. Patient Characteristics

This study included in total 141 plasma samples of 81 vulvar cancer patients and 60 healthy female donors. The mean age of the HDs was 59 years, with a range from 48 years to 74 years (medium: 59 years). The average age of cancer patients (*N* = 81) was 67 years, with a range from 28 to 93 years. The main histological subtype of vulvar cancers was VSCC (*n* = 78, 96.3%), and the majority of patients (*n* = 61, 75.3%) were included at their first diagnosis. The most frequent tumor stage was FIGO I (*n* = 40, 47.7%), followed by FIGO III (*n* = 32, 39.5%), FIGO IV (*n* = 10, 12.3%) and II (*n* = 1, 1.2%). Lymph node invasion was present in 37 cases (45.7%) and lymphangioinvasion in 22 patients (27.2%), and two patients had confirmed distant metastasis at the time point of sample acquisition, whereas the other eight patients in stage IV presented advanced local disease with extensive or ulcerated lymph node invasion. A surgical procedure was performed in 72 cases (88.9%), partially combined with adjuvant, neoadjuvant or concomitant radiochemo- or radiotherapy. Tissue HPV status could be obtained for 44 patients, of which 38 (86.4%) showed HPV-positivity. HPV16 was the predominant type (81.8%), and in 27.3% (*n* = 12), a multiple HPV infection was detected, mainly HPV45 (*n* = 7, 15.9%) and HPV31 (*n* = 6, 13.6%) (Table 1)

The clinical characteristics of the vulvar cancer patients are shown in Table 2.

### 3.2. NGS: Selection of Target exomiRs and Internal Control Genes

Using NGS, we identified seven single exomiRs capable of discriminating healthy individuals from vulvar cancer patients with high specificity. The analysis included raw NGS read counts, whole transcriptome quantification, exomiR annotation via miRBase and differential expression analysis. NormFinder output was used to assess expression stability, enabling the parallel identification of the most stable exomiRs suitable as internal controls for subsequent data normalization in the validation experiments with qPCR. Statistical evaluation applied stepwise False Detection Rate (FDR) values of *p* < 0.05 and *p* < 0.1 to prioritize robust candidates. The discriminative exomiRs exhibited significant fold changes (*p* < 0.05) across the dataset, while the stable reference exomiRs displayed minimal variability between groups. Based on this, the target exomiRs identified by NGS were: MiR-4516, miR-16-5p, miR-143-3p, miR-451a, miR-151a-5p, miR-223-3p and miR-12135 (more details in Supplementary File S3).

### 3.3. qPCR Validated exomiR Dysregulation in Vulvar Cancer

**Figure 1:**
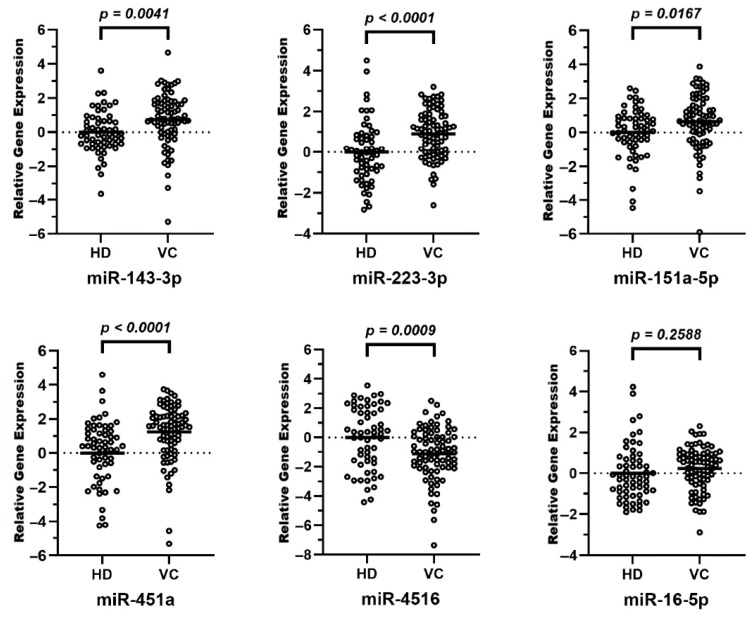
Validation of differential exomiR expression. Extracellular vesicle-related miRNAs were quantified using qPCR in a cohort of 81 vulvar cancer patients compared to 60 healthy controls. The raw Cq data were normalized to miR-378a-3p using the 2^−∆∆Ct^ method and subsequently transformed to log2 fold change, which is shown as relative gene expression, to allow for a symmetric visualization of up- and downregulation centered around zero. The bee-swarm plots show the relative gene expression of the 6 exomiRs, which were found significantly dysregulated in the plasma of cancer patients compared to healthy donors. Significantly upregulated were miR-143-3p, miR-223-3p, miR-151a-5p and miR-451a; significantly downregulated was miR-4516; no significant differential expression was shown for miR-16-5p.

**Figure 2:**
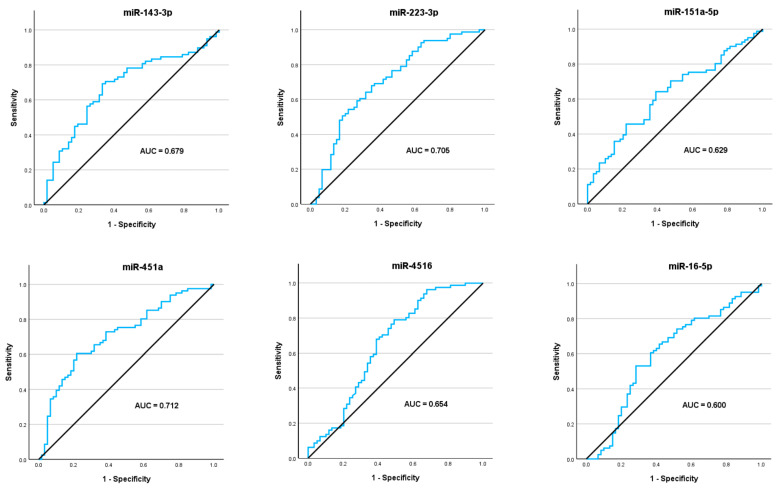
Evaluation of diagnostic accuracy of single exomiRs with ROC analyses. ROC curve analyses were performed to assess the diagnostic ability of each exomiR as an individual predictor for vulvar cancer. The AUCs of the single exomiRs ranged between 0.6 and 0.712. The equilibrium line shows performance relative to random chance.

The seven target exomiRs identified by NGS were analyzed using the miRCURY Custom PCR Panel. It validated that five of them were significantly dysregulated in plasma of vulvar cancer patients compared to the plasma of HDs. Four exomiRs were significantly upregulated: miR-143-3p, miR-223-3p, miR-151a-5p, and miR-451a, whereas miR-4516 was found to be significantly downregulated in the plasma of vulvar cancer patients compared to HDs. MiR-16-5p showed no significant differential expression. The AUCs of ROC curve analyses of the single exomiRs ranged between 0.6 and 0.712. (Table 3 and Figure 1 and Figure 2). MiR-12135 was not found to be expressed using this method, presumably due to very low expression level, which also explains the low NGS counts, and was thus excluded from further analysis.

### 3.4. Correlation Between exomiR Expression and Age

There was a significant age gap between VC patients and HDs (67 vs. 59 years). The Pearson correlation coefficient was calculated for all participants and in both cohorts—cancer patients and HDs separately—to rule out a confounding effect of age on subsequent analysis. We evaluated whether the age of the participants correlated with exomiR expression. Overall, no significant correlation was found (details available in Supplementary File S4), which suggests that the exomiR expression levels quantified in this study are not influenced by age. Differential gene expression between the two cohorts was tested with *t*-test and then additionally checked by analysis of covariance (ANCOVA) to adjust for age. Both tests delivered similar results for exomiR dysregulation without any significance of age, indicating that age was not a disturbing factor (Table 4).

### 3.5. Construction of a Combined exomiR Panel with Diagnostic Value

**Figure 3:**
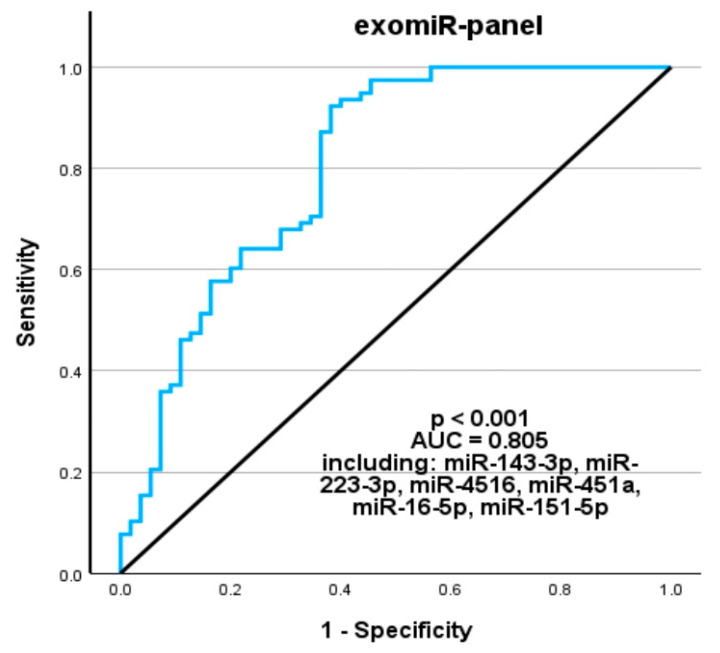
ROC curve analysis for the combined exomiR panel. Binary logistic regression was performed to identify an exomiR combination with highest diagnostic value. The best model included all 6 of the dysregulated exomiRs. The ROC curve for this panel was generated based on the predicted probability for each patient. Their combination resulted in a superior discriminatory ability compared to individual exomiRs. The term of the regression is: Logit(*p*) = −0.728 + 0.116 × miR-143-3p − 0.318 × miR-4516 + 1.012 × miR-223-3p + 0.619 + miR-451a − 0.268 × miR-151a-5p − 0.692 × miR-16-5p. The equilibrium line shows performance relative to random chance.

A binary logistic regression analysis was applied to construct a panel of multiple exomiRs, which showed superior discriminatory ability than each single exomiR (*p* < 0.001). The ROC-AUC of a panel consisting of all six exomiRs was considerably improved with 0.805 (95% CI: 0.726, 0.884; Sensitivity: 91%; Specificity: 61.8%; Youden’s Index: 0.528) (Table 3 and Figure 3).

### 3.6. Differential exomiR Expression Is Associated with Lymph Node Invasion

**Figure 4:**
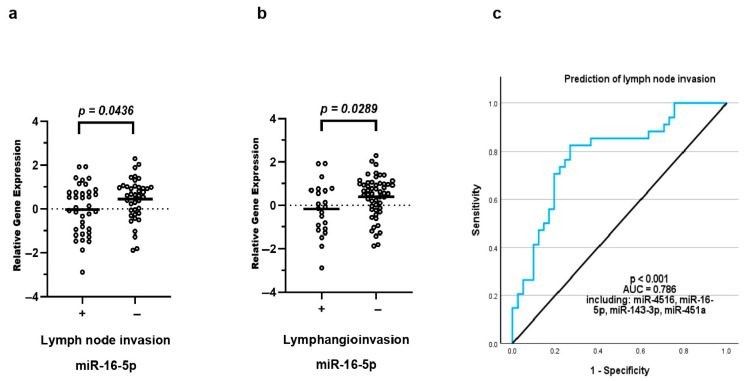
Differential exomiR expression and lymph node invasion. ExomiR expression levels were correlated with clinical parameters of vulvar cancer patients. Lower levels of miR-16-5p were observed in patients with lymph node positivity (**a**) and lymphangioinvasion (**b**). The combination of miR-16-5p, miR-4516, miR-143-3p and miR451a showed a moderate ability to discriminate nodal positive from nodal negative patients (**c**). The equilibrium line shows performance relative to random chance.

In a subgroup comparison between patients with lymph node metastasis (*n* = 37) and those without (*n* = 41), lower levels of miR-16-5p were observed in patients with lymph node positivity (*p* = 0.044; ROC-AUC = 0.623, 95% CI: 0.497, 0.749) (Figure 4). Similarly, decreased miR-16-5p expression was detected in patients with the presence of lymphangioinvasion (*n* = 22) compared to patients without (*n* = 53) (*p* = 0.029; ROC-AUC = 0.653, 95% CI: 0.516, 0.776; Figure 4). A binary logistic regression was conducted to identify a potential combination of exomiRs, which could predict lymph node positivity. The best model consisted of miR-4516, miR-143-3p, miR-16-5p, and miR-451a, showing a moderate discriminatory ability with a sensitivity of 70.6% and a specificity of 78% (*p* < 0.001, ROC-AUC = 0.786, 95% CI: 0.680, 0.891) (Figure 4).

### 3.7. Differential exomiR Expression Is Associated with Presence of Precancerous Lesions

**Figure 5:**
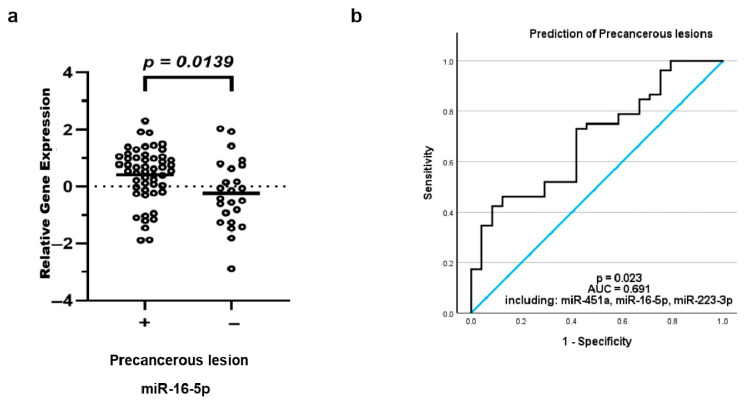
Differential exomiR expression and precancerous lesions. ExomiR expression levels were correlated with clinical parameters of vulvar cancer patients. Lower levels of miR-16-5p were observed in patients which presented a precancerous lesion, either in their history or concurrent to the vulvar cancer (**a**). The combination of miR-16-5p, miR-223-3p and miR451a showed a moderate ability to distinguish these two subgroups (**b**). The equilibrium line shows performance relative to random chance.

To investigate the discriminatory effect of exomiRs on VSCC patients with and without precancerous lesions, the cohort was subdivided into two groups: one with VIN or lichen sclerosus (*n* = 52) either already known prior to the tumor manifestation or diagnosed simultaneously during the assessment procedure; the other lacked any precancerous lesions, neither in their history nor in the surrounding areas of the carcinoma site (*n* = 24). A two-tailed *t*-test was conducted. In the precancerous lesion group, evident upregulation of miR-16-5p was observed compared to the non-precancerous group (*p* = 0.014, ROC-AUC = 0.674, 95% CI: 0.535, 0.813). Analysis of covariance showed that the upregulation was still independently statistically significant when adjusted for FIGO stage. Binary logistic regression showed that a combination of miR-16-5p, miR-223-3p and miR-451a had the potential ability to discriminate between patients with and without precancerous lesions with a sensitivity of 90.4%, but only a specificity of 25% (*p* = 0.023, ROC-AUC = 0.691, 95% CI: 0.567, 0.814; Figure 5).

### 3.8. Differential exomiR Expression Is Associated with HPV Status

**Figure 6:**
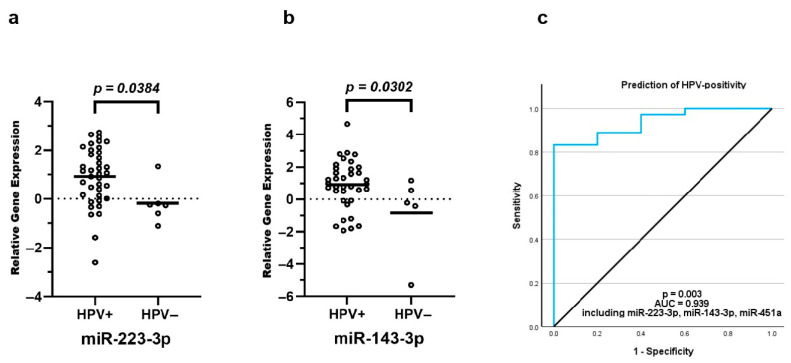
Differential exomiR expression and HPV positivity. HPV status was obtained using mass spectrometry and the results were correlated with exomiR expression. The expression levels of miR-223-3p and miR-143-3p were found elevated in patients with HPV-positivity (**a**,**b**). A binary logistic regression demonstrated that a model including miR-451a, miR-223-3p and miR-143-3p could predict HPV-positivity with an overall sensitivity of 97.2% and a moderate specificity of 60% (**c**). The equilibrium line shows performance relative to random chance.

Correlation of exomiR expression and HPV status suggested that expression levels of miR-223-3p and miR-143-3p were elevated in HPV-positive patients (*n* = 38) compared to HPV-negative patients (*n* = 6, *p* = 0.038 and 0.030; ROC-AUC = 0.785 and 0.761, 95% CI: 0.598, 0.972 and 0.577, 0.945). Binary logistic regression demonstrated that a model including miR-451a, miR-223-3p and miR-143-3p was a robust discriminator of HPV-positive and -negative groups with an AUC of 0.939, an overall sensitivity of 97.2% and a moderate specificity of 60% (*p* = 0.003, 95% CI: 0.859, 1.019) (Figure 6).

### 3.9. Correlation Between exomiR Expression and Clinical Characteristics

Taken together, exomiR expression was associated with the clinical parameter lymph node status, lymphangioinvasion, presence of precancerous lesions and HPV status. Given the exploratory nature of these analyses and the limited sample size for all subgroup analysis, but especially for the HPV-negative cohort, all findings should be interpreted with caution and considered as preliminary and hypothesis-generating.

### 3.10. Differential Expression of exomiRs Predicts Survival

**Figure 7:**
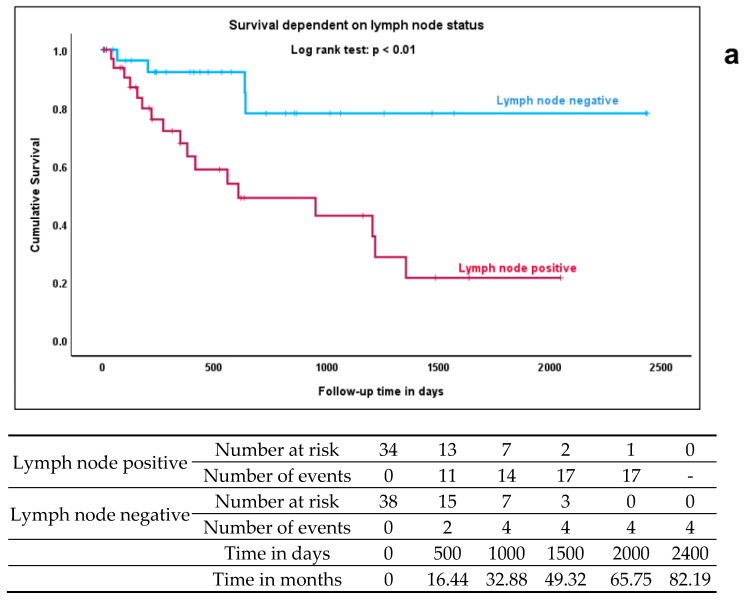

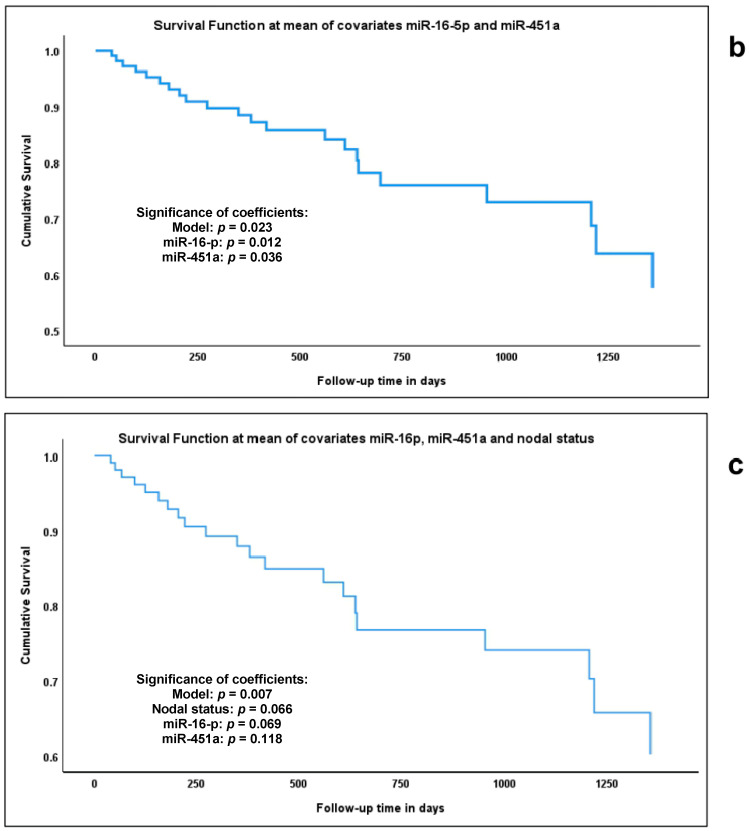
Lymph node metastasis and exomiR-expression are associated with worse overall survival. During the follow-up period, 22 patients died. Kaplan–Meier analysis showed that lymph node metastasis was a predictor of poorer survival (**a**). Multivariate Cox regression was performed to assess the impact of exomiR expression on survival. A model including the downregulation of miR-16-5p and simultaneous upregulation of miR-451a was associated with worse overall survival (**b**). When lymph node status was incorporated as well, the model still showed an overall significance, but the individual coefficients did not display statistical significance (**c**).

The median follow-up time was 526 days (17.3 months, IQR 70–725 days). The median survival time was 1223 days (40 months, IQR 139–891 days). Kaplan–Meier analysis with the log rank test showed that lymph node positivity was significantly associated with shorter overall survival (*p* < 0.01) (Figure 7a, Table 5). The respective survival table can be found underneath Figure 7a and in more detail in Supplementary File S5. Patients with lymph node metastasis had a median survival of 610 days (median not reached for patients without metastasis). Concomitantly, the FIGO stage was also associated with survival, since in our patient cohort- the FIGO stage was mostly determined by the lymph node status rather than by tumor size or extension. There was no association with HPV status or the administration of adjuvant or neoadjuvant therapy.

A multivariate Cox regression showed that a model of two covariates, including the downregulation of miR-16-5p and simultaneous upregulation of miR-451a, was associated with worse overall survival (model: *p* = 0.023), with both exomiRs as significant coefficients (miR-16-5p: *p* = 0.012, HR = 0.185, 95% CI: 0.05–0.687; miR-451a: *p* = 0.036, HR = 3.544, 95% CI: 1.088–11.542) (Figure 7b). When lymph node status was incorporated as well, the model still showed an overall significance of *p* = 0.007. Here, the individual coefficients did not display statistical significance (LN: *p* = 0.066, HR = 2.930, 95% CI: 0.930–9.230; miR-16-5p: *p* = 0.069, HR = 0.284, 95% CI: 0.073–1.102; miR-451a: *p* = 0.118, HR = 2.629, 95% CI: 0.782–8.833) (Figure 7c).

## 4. Discussion

Exosomal micro-RNAs are key regulators of tumor biology and have recently emerged as promising non-invasive biomarkers for diagnostic, prognostic and monitoring purposes, yet they have not been systematically investigated in vulvar cancer. Therefore, this study aimed to identify a potential diagnostic liquid biopsy marker for vulvar cancer through exomiR profiling. It was possible to establish an exomiR panel which could distinguish between vulvar cancer patients and non-affected women. The results at hand deserve special attention, since they proved the existence of exomiR dysregulation in vulvar cancer for the first time, yielding hope for new diagnostic possibilities. It is the first study which strives towards the detection and clinical utilization of miRNA-based liquid biopsy markers in this scarcely researched tumor entity, apart from the two studies analyzing cell-free (cf) miRNAs mentioned in the introduction. Not surprisingly, comparison of these previous findings with the exomiR profile detected in our study showed no overlap of dysregulated miRNAs, owing to the different release modalities: exosomal release might preferentially occur from certain tumor subclones, therefore carrying tumor-specific cargo, while total cf-miRNA reflects both tumor, stroma and possibly other cells causing divergences [86]. In addition, oncogenic signals like E6/E7 in HPV-associated cancers change cellular miRNA expression as well as exosomal secretion. Hence, the exomiR profile can diverge from the cell-free non-exosomal and intracellular miRNA profile [47].

Among all the exomiRs we analyzed, miR-16-5p is most frequently associated with other types of cancer, as reflected by the number of available corresponding publications. In most cancer entities studied so far, miR-16-5p predominantly plays a tumor-suppressive role, inhibiting carcinogenesis in vitro and in animal studies. It was found downregulated in tissue, peripheral blood and plasma exosomes of cancer patients, including ovarian cancer [87,88,89].

However, its differential regulation varies among different studies (see Supplementary File S1), indicating that its expression, and functional role, highly depends on the cancer entity and whether it is secreted as free-circulating miRNA, encapsulated into vesicles or found intracellularly.

In our subgroup analysis among cancer patients, miR-16-5p expression levels were inversely correlated with lymph node metastasis and lymphangioinvasion, which suggests that its expression and secretion might serve as protective factor against early tumor invasion. On the other hand, miR-16-5p expression was also found significantly increased in patients, which presented a premalignant lesion in their history, which might indicate that miR-16-5p is already upregulated during early stages of tumorigenesis. To assess whether the dysregulation of miR-16-5p could serve as an early marker for VIN progression to vulvar cancer, longitudinal studies in VIN cohorts are required.

MiRNAs are often classified as either oncogenic miRs or tumor-suppressor-miRs, depending on their overall regulation in cancers and their major effects in carcinogenesis, promoting or inhibiting growth. As visible with miR-16-5p, this definite binary classification is not always applicable, because miRNAs act as mediators in complex molecular networks, taking part in multiple pathways. Their ambivalent role depends on the functional context and is influenced by many internal and external co-factors, which are not fully disclosed yet [28,35,87]. The specific effects of miR-16-5p and the other analyzed exomiRs in vulvar cancer need to be investigated in future studies; the results of a literature review are described in the Supplemental Material (Supplementary File S1). The detailed understanding of the molecular mechanisms of actions and their functional relevance is especially important for their use as therapeutic targets or for the design of synthetic miRNA mimics as drugs.

To improve diagnostic accuracy while acknowledging the influence of confounding environmental factors and adjusting for interindividual variance of exomiR expression, randomized studies with clinically matched patients and controls are required. A biomarker panel combining multiple exomiRs is preferable to using a single exomiR. The ROC analysis in the present study showed that a panel consisting of six exomiRs outperformed the single exomiRs in their ability to discriminate vulvar cancer patients from HDs. Generally, AUC values above 0.8 are considered clinically useful [90], which attests the diagnostic power of the panel, proving the concept of our exomiR panel as a liquid biopsy tool for vulvar cancer in its current form, due to its limited specificity, primarily in pre-selected high-risk cohorts, e.g., HPV-positive women or women with pre-invasive lesions such as lichen sclerosus. Moreover, the combination of exomiRs with other biomarkers such as cf-miRNAs, circulating tumor cells (CTCs), ctDNA, proteins or other exosomal cargo could enhance the sensitivity and specificity of cancer detection [91,92], which is essential for its utilization as a screening method. Currently, no such biomarkers are available for vulvar cancer; however, once identified and characterized, they should be compared with cf-miRNAs and exomiRs to determine the most accurate and robust combination of analytes.

In our analysis, we identified two exomiRs whose expression levels were significantly associated with overall survival. Adjustment for lymph node status even increased the significance of the combined model while significance was lost for the individual exomiRs, suggesting that the impact of the exomiRs might be modulated by nodal status. Nevertheless, exomiR expression levels remain predictive of patient outcomes in the absence of lymph node information. This may have practical relevance in clinical scenarios where operative lymph node staging has not yet been performed or is not feasible due to the invasiveness and risk of the procedure. In such cases, exomiR profiling could provide useful prognostic information to guide early decision-making.

A potential limitation of our study is the age difference between vulvar cancer patients and the HD cohort. This discrepancy arises from the fact that blood samples of the control cohort individuals were obtained through the blood donor service, which imposes general health and age-related eligibility criteria. However, in the retrospective analysis, no correlation between age and the investigated parameters was observed, and the gene expression analysis showed significant results with and without age-adjustment. Therefore, it was deemed reasonable to analyze the data irrespective of age differences. However, in future studies, the healthy controls should preferably be age-matched to the cancer patients.

Furthermore, the interpretation of all subgroup analyses, but especially the correlation between HPV status and exomiR expression, should be approached with caution due to the very limited size of the HPV-negative test group, and further investigation is warranted.

It should be noted that the exomiR panel of this study is based on the analysis of a patient cohort restricted to women recruited in Germany. In its current form and yet limited specificity, it is not suitable as unselective and broadly applicable screening tool so far. For the clinical implementation of screening tools for rare diseases like vulvar cancer, efficiency and cost-effectiveness need to be evaluated, considering accuracy as well as the prevalence in the population and the expected benefit. For vulvar cancer, the assumed incidence is around 2–7/100,000 in Germany, with an estimated number of 3200 new cases annually [93,94]. For the early detection and diagnosis of diseases with such a low incidence, screening methods with nearly 100% sensitivity and specificity are warranted, for not missing out on any case but at the same time not causing a high number of false positive results. Our exomiR panel showed a sensitivity of over 90% with a moderate specificity of only 62%, which opposes its broad utilization as a primary screening tool. However, it could be used as an assessment tool in pre-selected groups, preferentially in addition to clinical evaluation and risk-stratification.

Certainly, an advantage of exomiR analysis is its non-invasive nature, enabling sequential testing, which is crucial for disease monitoring. However, there is a lack of standardized protocols for EV isolation, exomiR extraction and quantification, which leads to inconsistencies in findings across different studies and complicates their reproducibility [26,90]. A consensus on best practices for exomiR analysis is essential to ensure their validity. The standardization of technical procedures is the basis for reproducible and accurate results, facilitating the design of reasonable and feasible clinical applications which ensure long-term cost-effectiveness.

Upcoming evidence suggests that exomiRs are more sensitive, specific and reliable biomarkers for cancer diagnosis than cf-miRNAs, due to their selective enrichment into the EVs and their stability in circulation [36,95,96,97,98]. Their protection by lipid bilayers offers a significant advantage over cf-miRNAs and other biomarkers, which are susceptible to rapid degradation. Also, exomiRs are selectively sorted into the EVs and released intentionally by their cell of origin; therefore, exosome content reflects the status of the parental cell. The type and concentration of exosomes as such reflect the individual’s health status. The specificity of exosomal cargo enables the analysis of the exomiRs of interest even if they are low in total expression [95]. Various comparative studies support the superiority of exomiRs in cancer diagnosis in terms of their quantity, quality and stability [35,36,37]. An additional advantage is that the dysregulated expression of exomiRs can reflect the molecular characteristics of the tumor from which they originate, representing its genetic makeup and offering insight into tumor heterogeneity [39,99]. This could help to choose an individualized treatment option and adjust it precisely depending on the therapeutic response. For example, new tumor-targeted immunotherapies or antibodies could be adapted to molecular features, e.g., mutational status, of the tumor. However, it needs to be acknowledged that the current knowledge about the mechanisms of release, uptake and downstream effects of exomiRs is still very limited. Even though their value as circulating biomarkers is supported by accumulating evidence, statements about their functional implications should be interpreted cautiously. Technical challenges must also be considered, particularly the current lack of standardization and consistency of isolation methods, which leads to variability in RNA purity, quality, and concentration [39,80]. Also, most EV preparations represent a heterogeneous vesicle population originating from different biogenetic pathways, and strict post-release discrimination remains challenging.

As stated above, the methodology used in this study for EV purification and miRNA isolation have been proven by multiple preceding studies to deliver robust and reliable results [39,76,77,78,79,80,81]. In comparisons among six alternatives methods of EV-related RNA isolation, the exoRNeasy method showed the highest yield and a narrow distribution pattern, as well as the most uniform size of small RNA, indicating the highest extraction efficiency of small exoRNA from serum [80].

However, the absence of direct structural or functional characterization of the isolated EVs can be seen as a limitation of the present study. Although a standardized and widely used protocol was applied to enrich for small EV- and exosome-containing fractions, the lack of complementary analyses such as nanoparticle tracking analysis, electron microscopy, or EV marker profiling precludes the unequivocal attribution of the detected miRNAs to a specific EV subtype. Consequently, the observed miRNA signatures may reflect EV-associated miRNAs derived from a heterogeneous population of small EVs. Importantly, the primary aim of this exploratory, hypothesis-generating study was to assess the feasibility and potential clinical relevance of circulating EV-associated miRNA profiles as liquid biopsy biomarkers in vulvar carcinoma. In the context of clinical biomarker development, the central endpoint is the robust and reproducible quantification of marker candidates that discriminate between predefined clinical groups, while detailed functional and structural characterization, although highly desirable, often occurs in subsequent work. Our study follows this paradigm by concentrating on the quantitative assessment of candidate EV-associated miRNAs in relation to clinicopathological features in vulvar carcinoma, using a rigorously standardized pre-analytical workflow from blood collection tube, sample handling and storage to isolation protocol, assay performance and data evaluation [100]. The explicit reporting of a highly standardized, uninterrupted end-to-end isolation workflow contributes to technical transparency and reproducibility. Such robust one-step protocols may represent a practical advantage for quantitative biomarker applications, where pre-analytical consistency and analytical reliability are critical determinants of clinical utility. Still, future prospective studies are desirable to integrate systematic EV-phenotyping alongside miRNA profiling to further refine vesicle attribution and strengthen biological interpretation.

In summary, our study indicates that the development of liquid biopsy-based exomiR analysis holds big promise for diagnosis and disease monitoring in the modern clinical management of vulvar cancer, being non-invasive, stable and disease-specific. The significant dysregulation of several exomiRs compared to healthy controls as well as in subgroup analysis strongly supports the hypothesis that exomiRs are not only powerful diagnostic markers but may also be suitable for risk stratification and the assessment of HPV, which could be critical for therapeutic decisions and follow-up care. It is necessary to validate our findings in larger, preferably age-matched multi-center studies with a long-term follow-up to disclose clinical effectiveness and applicability for disease diagnosis, therapy monitoring and risk stratification.

In the future, the integration of exomiRs with other biomarkers could enable a multimodal approach to tumor heterogeneity, allowing for the adaptation of tumor-tailored treatment. In the era of targeted therapies, such as immunotherapies that focus on the genetic characteristics of cancer, molecular markers play a crucial role in selecting the appropriate drug, predicting treatment response, and assessing resistance. In high-risk groups they might be used as screening tools to monitor progression to cancer, and to enable early detection of clinically inapparent tumors or minimal residual disease after treatment. In combination with regular gynecological check-ups, they could improve the early diagnosis and management of vulvar cancer and its premalignant lesions. Future longitudinal studies that include patients with precancerous lesions are required to determine whether exomiR dysregulation is also present in VIN patients and whether exomiRs can predict malignant transformation and progression to invasive carcinoma. In addition, assessment of temporal changes in exomiR expression will uncover their suitability as monitoring biomarkers.

Overall, liquid biopsy techniques are a step towards a personalized medicine and precision oncology [101,102,103]. Challenges related to technical issues and the complexity of exomiR biology and their functional role in cancer need to be addressed by future research.

## 5. Conclusions

In summary, our study indicates the future potential of circulating exomiRs as liquid biopsy markers in vulvar cancer, which is a first step towards the implementation of a noninvasive screening tool and can hopefully contribute to draw more attention to this rare disease. This is also a step-one study paving the way for a predictive risk-stratification tool for VIN patients.

## Figures and Tables

**Table 1:** HPV status of tumor tissue.

HPV Status Assessed In	44
Positive	38 (86.4%)
Multiple HPV infection	12 (27.3%)
HPV16	36 (81.8%)
HPV31	6 (13.6%)
HPV45	7 (15.9%)
Negative	6 (13.6%)

The table shows the results of the HPV assessment of the tumor tissue using mass spectrometry.

**Table 2:** Clinical characteristics of cancer patients.

Number of Vulvar Cancer Patients	81
Mean age (range) {median} of cancer patients	67 (28–93) {68}
Diagnosis	
Vulvar squamous cell carcinoma	78 (96.3%)
Adenosquamous carcinoma	1 (1.2%)
Basal cell carcinoma	1 (1.2%)
No further specification	1 (1.2%)
FIGO Stage	
I	40 (47.7%)
II	1 (1.2%)
III	32 (38%)
IV	10 (11.9%)
No data *	1 (1.2%)
Lymph node invasion	
positive	37 (45.7%)
negative	41 (50.6%)
No data *	3 (3.7%)
Lymphangioinvasion	
positive	22 (27.2%)
negative	53 (65.4%)
No data *	6 (7.4%)
Distant metastasis	2 (2.5%)
Time point of sample collection	
First diagnosis	61 (75.3%)
First relapse	9 (11.1%)
Second or more relapse	10 (12.3%)
No data *	1 (1.2%)
Treatment (after enrolment)	
Surgery only	43 (53.1%)
Combined surgery and chemotherapy **	2 (2.5%)
Combined surgery and radiochemotherapy ***	14 (17.3%)
Combined surgery and radiation	12 (14.8%)
Primary or palliative radiochemotherapy	6 (7.4%)
Best supportive care	2 (2.5%)
No data *	1 (1.2%)
Precancerous lesions present	52 (64.2%)

* For reasons of statistical clarity, missing data is reported as part of 100%. Missing data points were handled with pairwise deletion during statistical analysis. Patients with missing data for one category were still included in every other subgroup analysis where data is available. ** one of these patients started a neoadjuvant chemotherapy prior to enrolment. In this case blood sample collection was done before surgery three months after the end of the chemotherapy. *** two of these patients started a neoadjuvant radiochemotherapy prior to enrolment. Blood samples were taken before surgery.

**Table 3:** Differential exomiR expression without adjustments.

exomiR	Fold Change Ratio	*p*-Value	ROC-AUC	95% CI	Youden Index	Sensitivity	Specificity
miR-143-3p	1.66	0.004	0.679	0.587, 0.770	0.359	0.692	0.667
miR-223-3p	1.31	<0.001	0.705	0.616, 0.793	0.327	0.543	0.783
miR-151a-5p	1.71	0.017	0.629	0.537, 0.721	0.236	0.457	0.78
miR-451a	1.75	<0.001	0.712	0.626, 0.798	0.376	0.593	0.783
miR-4516	0.39	<0.001	0.654	0.558, 0.750	0.299	0.508	0.79
miR-16-5p	0.83	0.258	0.6	0.502, 0.698	0.238	0.605	0.633
exomiR panel		<0.001	0.805	0.726, 0.884	0.528	0.91	0.618

The table lists the fold change ratio of the exomiR expression levels of vulvar cancer patients compared to healthy controls after normalization. The *p*-value was calculated with *t*-tests for the individual exomiRs and with Chi-square tests for the panel. The AUCs derived from ROC curve analyses are shown together with corresponding 95% CI.

**Table 4:** Age-adjusted differential exomiR expression.

exomiR	Mean Difference (VC–HD)	*p*-Value	95% CI
miR-143-3p	0.843	0.002	−1.371, −0.315
miR-223-3p	0.978	<0.001	−1.451, −0.505
miR-151a-5p	0.663	0.019	−1.214, −0.112
miR-451a	1.151	<0.001	−1.791, −0.510
miR-4516	−1.346	<0.001	0.698, 1.995
miR-16-5p	0.201	0.345	0.621, 0.219
exomiR panel			

The table shows the results of the age-adjusted comparison of the exomiR expression in vulvar cancer patients compared to healthy controls, which was calculated using univariate analysis of covariance (ANCOVA).

**Table 5:** Kaplan–Meier analysis by lymph node status.

Nodal Status	Total N	N of Events	Censored-N	Censored-Percent
Negative	39 *	4	35	89.7%
Positive	35 *	17	18	51.4%
Overall	74 *	21	53	71.6%

* The divergence in numbers compared to Table 2 is due to missing PCR data points.

## Data Availability

The datasets generated and analyzed during the current study will be uploaded to Zenodo (DOI 10.5281/zenodo.18266416); additional information is available as Supplementary Material (Supplementary Files S3 and S4).

## Supplementary Materials

The following supporting information can be downloaded at: https://www.mdpi.com/article/10.3390/cancers18030438/s1, Supplementary File S1: Synopsis of literature research; Supplementary File S2: Materials and methods—Detailed description of the workflow; Supplementary File S3: Results of next-generation sequencing—Overview of the most dysregulated exomiRs; Supplementary File S4: Analysis of the correlation between exomiR expression and age of participants; Supplementary File S5: Survival table—Kaplan–Meier analysis by lymph node status. References [52,53,54,55,56,57,58,59,60,61,62,63,64,65,66,67,68,69,70,71,75] are cited in the Supplementary Materials.

## Abbreviations

The following abbreviations are used in this manuscript:

AUC	Area under the curve
cDNA	Complimentary DNA
cf-miRNA	cell-free miRNA
CI	Confidence interval
ctDNA	Circulating tumor DNA
DNA	Desoxyribonucleic acid
EV	Extracellular vesicle
exomiR	exosomal micro-RNA; EV-associated micro-RNA
FFPE	Formalin-fixed paraffin-embedded
FIGO	Fédération Internationale de Gynécologie et d’Obstétrique/International Federation of Gynecology and Obstetrics
FUP	Follow-up period
HD	Healthy donor
HPV	Human papillomavirus
miR/miRNA	Micro-RNA
NGS	Next-generation sequencing
PCR	Polymerase chain reaction
qPCR	Quantitative, real-time PCR
RNA	Ribonucleic acid
ROC	Receiver operating characteristics
RT	Reverse transcriptase
SCC	Squamous cell carcinoma
VSCC	Vulvar squamous cell carcinoma
