# Supplementary material for: Liquid Biopsy Analysis of the EV-Associated Micro-RNA Signature in Vulvar Carcinoma May Benefit Disease Diagnosis and Prognosis
Source: Cancers (Basel). 2026 Jan 29;18(3):438. doi: 10.3390/cancers18030438 (PMC12896608; doi:10.3390/cancers18030438)
Supplement: Supplementary file 1 [file cancers-18-00438-s001.zip › Supplementary File S1.pdf]

Supplementary Material S1 – Synopsis of literature research

miR-12135: There were no reports on exosomal miR-12135 and in general, only one sole study was found which mentioned this miRNA at all, in the context of liver fibrosis [83].

miR-4516: Exosomal miR-4516 was found increased in the plasma of small cell lung cancer patients. The treatment with a gapmer antisense oligonucleotide (gASO) targeting Serin/Arginin repetitive matrix 4 (SRRM4), a splicing activator, suppressed the tumor in mouse models and lead to a reduced plasma level of miR4516. This first approach to miRNA-based therapeutics and disease monitoring purposes indicates its future potential in clinical applications [84].

miR-151a-5p: The differential regulation of circulating exosomal miR-151a-5p has already been studied in several diseases, tumorous as well as non-tumorous entities. For example, it was found dysregulated in endometrial cancer [85] and lung cancer [86, 87]. However, its functional role is not yet disclosed.

miR-16-5p: MiR-16-5p and exosomal miR-16-5p have been mentioned in diverse contexts. Interestingly, they seem to be involved in regulation and molecular pathways of Programmed death-ligand 1 (PD-L1). In lung adenocarcinoma, exosome-derived miR-16-5p has been found downregulated in patient serum and cell culture medium and it was linked inversely to tumor stage and PD-L1 expression. Also, exosomal miR-16-5p overexpression in a cell culture medium decreased PD-L1 expression and hence inhibited the tumor formation, which suggests that miR-16-5p is a latent tumor suppressor and might be utilized as immunotherapy biomarker [68]. These findings are supported by an in vitro experiment, which showed that miR-16-5p was transferred intercellularly via exosomes from macrophages to gastric cancer cells, where it decreased the expression of PD-L1. Subsequently it triggered an immune response, which suppressed carcinogenesis. This effect was reproduced in mice models [88]. In South African population, the ratio of exosomal miR-194-5p/miR-16-5p could be used as a non-invasive marker for the evaluation of prostate cancer aggressiveness [89]. As part of a multi-biomarker panel, serum exosomal miR-16-5p could be also used to distinguish esophageal adenocarcinoma from controls and Barrett's esophagus [90].

Interestingly, a study investigating exomiRs as biomarkers for cervical cancer, found exosomal miR-16-5p stably expressed in cancer patients and healthy controls and even used it for normalization [91]. This study used a similar design to our study, performing NGS first and secondly qPCR. Exosomal miRNA was isolated using Qiagen kits. Due to technical comparability (utilization of similar exomiR purification protocols), their results should be considered carefully. Also, nanoparticle tracking analysis and transmission electron

microscopy was conducted to control the success and quality of exosome isolation, which again proofs that Qiagen kits are eligible for exosome purification.

Moreover, miR-16-5p has been found dysregulated in multiple solid tumors, in tissues as well as in serum, and its function and shifts in anti-tumor treatment have been already studied and reviewed. The expression pattern in tissue of diverse cancer entities suggests a putative tumor-suppressive effect and indicates that it could serve as promising biomarker and therapeutic target [67, 92]. However, there is limited data about its presence in exosomes and its functional role as well as its involvement in downstream signaling pathways, which impedes to predict its implications in the tumorigenesis of vulvar cancer.

miR-143-3p: The dysregulated expression of miR-143-3p is associated with various tumor diseases. For example, exosomal miR-143-3p is overexpressed in serum of endometrial cancer and ovarian cancer patients and could function as liquid biopsy marker, in combination with 5, respectively 4 other miRNAs [93, 94]. Interestingly, the tissue expression of miR-143-3p in endometrial cancer tissue was upregulated, concordant to the serum expression. In contrast, it was downregulated in ovarian cancer tissue, discordant to ovarian cancer serum. Amongst others, exosomal miR-143-3p has also been mentioned in connection with colorectal cancer [95], as well as it was identified in urinary exosomes as a marker for prostate cancer [96].

However, another group found that miR-143-3p downregulated in ovarian cancer tissue and hypothesized its function as a tumor suppressor, as they also showed that its upregulation in cell lines reduced proliferation, migration and invasion [97].

miR-223-3p: Exosomal miR-223-3p is mentioned in relation to various cancers, e.g. its expression level in plasma is elevated in breast cancer patients with invasive ductal carcinoma when compared to patients suffering from ductal carcinoma in situ [98, 99].

miR-451a: Exosomal miR-451a expression has already been investigated in multiple cancer entities and interestingly sometimes found to be up- and sometimes downregulated which points towards an ambiguous functional role. In pancreatic cancer, miR-451a showed an excellent diagnostic power and significant association to clinical stage and metastasis when overexpressed [100]; in non-small cell lung carcinoma its upregulation was positively correlated to recurrence, stage and lymph node metastasis and it is hence proposed to serve as biomarker for the prediction of recurrence and prognosis [101]. In contrast, in plasma of hepatocellular carcinoma (HCC) patients, miR-451a was found downregulated and in-vitro trials showed its ability to induce apoptosis both in HCC cell lines and human umbilical cord vein endothelial cells, indicating its tumor-suppressive role [102].
