# Supplementary material for: Liquid Biopsy Analysis of the EV-Associated Micro-RNA Signature in Vulvar Carcinoma May Benefit Disease Diagnosis and Prognosis
Source: Cancers (Basel). 2026 Jan 29;18(3):438. doi: 10.3390/cancers18030438 (PMC12896608; doi:10.3390/cancers18030438)
Supplement: Supplementary file 1 [file cancers-18-00438-s001.zip › Supplementary File S2.pdf]

Supplementary File S2 – Material and Methods – Detailed Description of the workflow

EV and RNA isolation

Prior to the RNA isolation, the plasma was pre-filtered using Millipore Millex AA Syringe filter (Merck Millipore, Burlington, MA, USA), with a pore size of 0.8 µm, according to manufacturer's recommendation, and diameter ø 33 mm, to eliminate larger particles and the platelets [52-54].

The purification of the exosomal RNA was performed using the exoRNeasy Midi Kit (Qiagen cat. No.77144, Hilden, Germany). The workflow was executed according to the manufacturer's instructions. The starting plasma volume was 800 µl.

Part number one of the protocol consisted in the isolation of the extracellular vesicles. First, 800µl of buffer XBP were added to the plasma. The solution was mixed by inverting repeatedly before it was added to the exoEasy-spin column device (Qiagen, Hilden). It was centrifuged at 500 x g für 1 min, the flow-through was discarded and 3.5 ml of XWP buffer was added, followed by another centrifugation at 5000 x g for 5 min. Hereafter, the spin column was transferred to a fresh collection tube and the extracellular vesicles were washed out from the membrane by adding 700 µl Qiazol lysis reagent and spinning for 5 min at 5000 x g.

Part number two of the procedure consisted in the isolation and elution of the exomiRs: The lysate from part number one was vortexed and incubated at room temperature for 5 min. Meanwhile, the RNA spike-in controls were added, which comprised a template mix of UniSp2, UniSp4 and UniSp5 and the cel-miR-39-3p. Next, 90 µl chloroform were added and the tube was shaken vigorously for 15 s. After another incubation for 2 - 3 minutes, the solution was centrifuged for 15 min at 12000 x g and at 4 °C degrees. This step led to a phase separation with 3 phases: an upper, colourless aqueous phase containing RNA; a thin, white interphase; and a lower, red organic phase. The volume of the aqueous phase comprised approximately 400 µl and was transferred to a new collection tube. 800 µl of 100% ethanol were added and the solution was mixed thoroughly by pipetting up and down. The sample was then pipetted into the RNeasy MinElute spin column and centrifuged for 15 s at 8000 x g at room temperature. The MinElute spin column has a maximum binding capacity of 45 µg RNA, according to the manufacturer's information. 700 µl of the RWT buffer were added and the column were spun again. Afterwards, 500 µl of the RPW buffer were added followed by another centrifugation for 15 s at 8000 x g. This step was repeated with a spinning time of 2 min. The flow-through in the collection tube was discarded after each step.

Afterwards the MinElute spin column was placed in a new collection tube and centrifuged with open lid for 5 min to dry the membrane. The collection tube was then replaced again, and the remaining RNA was eluted by adding 140 µl of RNAse free water and spinning at full speed for 1 minute. The eluate was stored at -80 °C until further utilization.

RT - cDNA Synthesis

For the synthesis of the corresponding cDNA, a reverse transcription of the isolated RNA was performed using the miRCURY LNA RT-kit (Qiagen, cat. No. 339340, Hilden, Germany). All the RT reactions were set up on ice. The total reaction volume was 10 µl which contained the initial template RNA volume of 1.4 µl. This volume was calculated using the recommended calculation method by Qiagen for the estimation of the RNA amount in the solution.

Template RNA [µl] = Elution volume [µl] / Original sample volume [µl] * 8 [µl]
= 140 µl / 800 µl * 8 µl = 1.4 µl

Besides the template RNA, the reaction mix consisted of the following components: 5x miRCURY SYBR Green reaction buffer, RNase-free water, 10x miRCURY enzyme mix and the UniSP6 spike in control. The reaction mix was prepared and then incubated for 60 min at 42 °C degrees, Following the manufacturer's manual for first-strand cDNA synthesis. Afterwards, it was heated to 95 °C degrees for 5 min to heat-inactivate the reverse-transcriptase and then immediately cooled to 4 °C degrees. cDNA was stored at -15 °C to -30°C for a maximum of 5 weeks until further utilization.

The synthesized cDNA was then used for qPCR applying the miRCURY custom panels. For detailed information regarding the components of the reagents, please consult the manufacturer's handbooks and product specifications.

qPCR – Quality control

Before running the qPCR Custom panels, each sample was subjected to a quality control using the miRCURY LNA miRNA QC PCR Panel (cat. no. 339331). Each panel comprised 96-wells with 12 different assays configured for 8 individual samples. The process protocol for the QC-panel was the same as for the customized panel, with a total input amount of 2.5 µl of diluted cDNA per sample, which corresponded to a volume of 0.2 µl of cDNA per assay.

It served to observe the process performance and technical execution of the RNA purification as well as to assess the biological quality of the samples. Therefore, it included UniSP3, which served to monitor the PCR efficiency. It also comprised primers for both, UniSP6 and cel-miR-39, to evaluate efficiency of cDNA synthesis and primers for UniSP2, UniSP4 and UniSP5. These Spike-in controls served to assess the success of the RNA isolation. Since they were present at a 100-fold concentration in the solution added during RNA purification process, it resulted in a difference of Cq-values in a range of 5 - 7 which hence served to control accuracy of RNA purification.

Furthermore, the QC-panel included two assays for human miRNAs, which are known to be stably expressed in blood, namely miR-23a-3p and miR-451a. MiR-23a-3p which is known to be stably expressed in blood and not affected by haemolysis. On the other side, miR-451a is known to be also abundantly expressed in blood but mainly derived from red blood cells. In the case of haemolysis, its expression level would raise in the samples, due to the damage of the erythrocytes. Hence, an increasing difference in Cq-values of both miRs at the same time can be used as parameter to monitor possibly haemolytic samples [103].

All samples passed this quality control and were subsequently admitted to the following analysis with the customized panel.

The cycler used for all PCR reactions was BioRad CFX, performing dye-based PCR with SYBR Green and using the 2x miRCURY SYBR Green master mix. The cycler algorithm was programmed the following way: The polymerase initial heat activation took place at 95 °C degrees for 2 min, followed by a 2-step cycle sequence which consisted of the denaturation at 95 °C degrees for 10 s and the combined annealing and extension phase at 56 °C degrees for 60 s. Both steps were repeated for a total of 40 cycles. The data acquisition was performed at the end of every cycle.

qPCR - Custom panel and normalization

Quantitative Real-Time PCR was performed using the miRCURY LNA miRNA Custom PCR Panel (cat. nos. 339330, 339332, Qiagen, Hilden, Germany) and the miRCURY LNA SYBR

Green PCR Kit (cat. no. 339347). Each 96-well panel was configured for 8 samples and 12 assays per sample. For each sample it contained primers for the 7 selected target-microRNAs and 4 of the selected endogenous controls, the microRNAs which should be used for later normalization. Additionally, for each sample one well was reserved for an interplate calibrator. For each well, a template volume of 0.625 µl of cDNA was used. This volume was determined after conducting several test runs to obtain Cq-values in an optimal range between 20 and 30.

For the miRCURY Custom panel, UniSP3 was used to monitor the PCR efficiency. The cycler algorithm was programmed the same way as for the QC protocol (see description above).

Single assay

MiR-12135 was not detected at the Custom panels, assumably because its concentration was too low. Therefore, a single assay was employed, to be able to process higher input concentrations of RNA and cDNA input. For this assay, miRCURY LNA miRNA PCR Assay (Cat. No. 339306, Qiagen, Hilden, Germany) was used, performing the same cycling steps described above. UniSP6 served as interplate calibrator and hsa-miR-378a-3p was used for normalization of data.

For the single assays, the cDNA synthesis was conducted with the same protocol as the custom Plates (see description above), but with an initial RNA template volume of 4.2 µl. This higher volume was set since the expected amount of the target Micro-RNA was only very low and it was not possible to detect it with lower input volume. The PCR assay was then run with an input cDNA amount of 0.625 µl, following the same cycling protocol as described above for the customized panels.

For detailed information regarding the ingredients of the reagents, please consult the manufacturer's manuals and Qiagen handbooks.

DNA isolation from FFPE tissue

Vulvar carcinoma samples were acquired postoperatively by the Institute of Pathology of the University Hospital Hamburg Eppendorf for all patients, who underwent surgery at the UKE. The carcinoma tissue, biopsy material or lymph node samples were obtained, processed with a histopathological work-up and were then stored in form of formalin-fixed paraffin-embedded (FFPE) blocks. 44 of them were provided for the MassArray analysis.

To analyse the HPV presence in the tissue with mass spectrometry, the DNA needed to be extracted. Before DNA extraction, each sample was visually checked for the presence of tumorous parts in it by microscopy.

50mg of each sample was dissected from the paraffine-embedded block, transferred into a tube and deparaffinated by the following protocol: It was twice incubated at room temperature in ethanol absolute for 5 min, then centrifuged at 12000 x g and the supernatant was discarded. The pellet was dried and then incubated with1 ml xylol for 10 min at 65 °C. Again, it was centrifuged for 1 min at 12000 x g, and the procedure was repeated, first with 1 ml of xylol at 65 °C, then with ethanol absolute at room temperature. The pellet was then incubated for 20 min at room temperature with ethanol 90%, centrifuged, dried and the supernatant was discarded. This procedure was repeated with ethanol 80%.

To extract the DNA of the FFPE tissue, the black PREP FFPE DNA Kit by Analytik Jena GmbH&Co KG was used and the procedure was performed following the manufacturer's instruction (Publication No.: HB_BP-0021_e_180808 1). First, for the lysis of the DNA, the tube containing the sample was centrifuged at 12000xg and 400 µl of Lysis Solution MA and 40 µl

of Proteinase K was added. The tube was vortexed for 10 s and then incubated with continuous shaking in a pre-heated thermal mixer: First, for a minimum of 12 hours at 65 °C, secondly for one hour at 90 °C and thirdly for 5 min at room temperature. It was centrifuged at 12000 x g for 2 min and for the next step, the binding of the DNA, the sample was transferred to a new receiver tube with 1.5 ml volume. Then, 400 µl ethanol absolute was added, it was mixed through pipetting up and down and the spin filter was added to the receiver tube. The sample was added to the spin filter, and it was centrifuged at 10000xg for 1 min. Then, 500 µl Washing Solution C was added, it was spun at 10000 x g for 1 min. After that, 650 µl Washing Solution BS was added, it was again spun at 10000 x g for 1 min and lastly 650 µl of ethanol absolute was added and it was centrifuged at 10000 x g for 1 min. To remove the ethanol, the spin filter was added to a new receiver tube whilst the filtrate was discarded. It was then centrifuged at 12000 x g for 3 min and the spin filter was added to the elution tube. For DNA elution, 75 µl of elution buffer was added and it was incubated for 2 min at room temperature, before it was centrifuged at 10000 x g for 1 min.

After the elution, the concentration of DNA in each eluate was determined with Qubit 4 and the Invitrogen Qubit dsDNA HS-Assay (Thermo Fisher Scientific), which is a fluorometric quantification method. Therefore, 1 µl of each sample was analysed. The purified DNA was stored at -20 °C until further use.

MassArray – HPV Genotyping Agena Bioscience

The HPV measurement was conducted using the Agena Bioscience HPV Genotyping Panel v.2.0 Agena on the MassARRAY® System (MassArray Analyzer 4, Agena Bioscience, San Diego, USA), which is a matrix-assisted laser desorption ionization time-of-flight (MALDI-TOF) mass-spectrometer (Vanessa). The panel is a single well assay, which is able to detect 24 different HPV subtypes in one multiplex assay, including 12 high-risk, carcinogenic subtypes, 1 probably carcinogenic, 7 possibly carcinogenic, and 4 low risk, not carcinogenic subtypes as well as an internal control (GAPDH), to confirm the presence of DNA in the reaction. An overview of the HPV types included can be found in the manufacturer's protocol. For the assay panel, premixed HPV PCR primers were used, which are based on type-specific sequences in the genomic E6/E7 region for 24 types.

The MassArray launches an automated report from within the MassArray Typer software and provides all positive and negative HPV types. The panel contained an internal process control targeting GAPDH in each reaction. Additionally, the panel contained four negative controls of water and two positive controls, consisting of DNA obtained of a cervical cancer cell line (Hela cell line), which is positive for HPV18.

The MassArray protocol comprises 3 steps, first a PCR amplification, secondly the shrimp-alkaline-phosphatase (SAP) reaction, thirdly the iPLEX Pro Extension Reaction. All reactions were set up and performed following the manufacturer's instructions (Agena (ABA) HPV GT Panel v2.0, (#06160) protocol). For detailed information regarding the ingredients of the reaction cocktails, see the manufacturer's handbook and the tables below.

According to the standard protocol, the MassArray requires an input amount of DNA between 5ng and 50ng per well, which correlate to a recommended DNA input volume of 2µl with a minimal concentration of 2.5ng/µl (PMID: 35123039). Wherever the concentration exceeded this amount, a working solution was diluted to 25ng/µl. All samples, where the concentration of DNA was lower than 3.5ng/µl were proceeded with a pooling protocol, which is already established for HPV detection in cervical carcinoma [61]. Therefore, for each sample a quadruplicate of the initial multiplex PCR was conducted, with 4 parallel reactions per sample

containing 2.5 µL of DNA and 3 µL of PCR mix. For the SAP reaction, the 4 wells were pooled, which resulted in a (total volume of 22 µL and in addition, 8 µL of the SAP mix. After the SAP reaction, 7 µL of the mix were combined with 2 µL of the extension mix and further processed according to the standard protocol.

The reaction mixes for the MassArray Protocol were prepared as master mix cocktails with a safety addition of 25% and then dispensed to each well, with a reagent's volume according to the table below.

First, 2.5 µl of the sample DNA was dispensed to a reaction plate, for the samples, who were low in DNA concentration, 4 wells were filled. The plate was sealed and centrifuged for 1 minute at 2000 x g. The PCR cocktail was prepared in a tube placed on ice by adding the reagents listed below. The tube was vortexed and briefly centrifuged and 3 µl of the cocktail was added to each well of the reaction plate. The plate was sealed, again vortexed and centrifuged and thermocycled using the following protocol:

1. 10 min at 30 °C
2. 2 min at 95 °C
3. Repeat for 10 cycles:
   3.1 30 s at 95 °C
   3.2 30 s at 60 °C
   3.3 1 min at 72 °C
4. Repeat for 35 cycles:
   4.1 30 s at 95 °C
   4.2 30 s at 56 °C
   4.3 1 min at 72 °C
5. 5 min at 72 °C
6. Hold at 10 °C

After the PCR amplification reaction, the samples, which were subjected to the pooling protocol, were merged into one single well.

The SAP cocktail was prepared on ice by adding the reagents as listed below. The tube was vortexed and centrifuged, as well as the reaction plate. For the pooled samples, 8 µl of the cocktail was added, for all other samples, 2 µl were dispensed into each well. The plate was sealed, vortexed, centrifuged and thermocycled using the following conditions:

1. 40 min at 37 °C
2. 5 min at 85 °C
3. Hold at 10 °C

It was then proceeded to the iPlex Pro Extension Reaction. The iPlex reaction cocktail was prepared in a tube placed on ice by adding the reagents according to the table below. The tube was briefly vortexed and centrifuged.

The reaction plate was centrifuged. From every pooled assay, 7 µl were transferred into a new, empty well. The residue was discharged. 2 µl of the iPlex cocktail mix was added to each pooled sample, 1.5 µl was added to each of the not pooled sample. The reaction plate was sealed, vortexed and centrifuged and then thermocycled according to the following protocol:

1. 30 s at 95 °C
2. Repeat for 40 cycles:
   2.1 5 s at 95 °C
   2.2 Repeat for 5 cycles

    2.2.1 5 s at 52 °C
    2.2.2 5 s at 80 °C
3. 3 min at 72 °C
4. Hold at 10 °C

After the extension reaction, 41 µl of HPLC grade water was added to each well. The plate was sealed and centrifuged for 1 min and then processed with the MassArray System with the Chip Prep Module. It was used the instrument's setting for iPlex Pro Genotyping and the MassArray software then launched an automated report that contained all positive and negative HPV types. The acquired data was analysed using the MassArray Typer v4.1.83.

Reaction cocktail mix:

Reagent	Per well in µl
Water	0.3
10x PCR buffer	0.5
MgCl2	0.4
dUTP/dNTP mix	0.1
PCR enzyme	0.2
HPV panel primer mix	1.0
UNG enzyme	0.5
PCR cocktail final volume	3
DNA input	2.5
PCR reaction final volume	5.5

Table 1 PCR Amplification reaction cocktail

Reagent	Per well in µl
Water	1.53
10x SAP buffer	0.17
SAP enzyme	0.3
Final volume SAP cocktail	2/8

Table 2 SAP reaction cocktail

Reagent	Per well in µl – not pooled assays	Per well in µl – pooled assays
Water	0.12	0.62
iPlex buffer plus	0.2	0.2
iPlex termination mix	0.2	0.2
iPlex pro enzyme	0.04	0.04
HPV genotyping panel extend primer mix	0.94	0.94
Extension cocktail final volume	1.5	2

Table 3 IPlex Pro Extension reaction cocktail
