# Supplementary material for: Liquid Biopsy Analysis of the EV-Associated Micro-RNA Signature in Vulvar Carcinoma May Benefit Disease Diagnosis and Prognosis
Source: Cancers (Basel). 2026 Jan 29;18(3):438. doi: 10.3390/cancers18030438 (PMC12896608; doi:10.3390/cancers18030438)
Supplement: Supplementary file 1 [file cancers-18-00438-s001.zip › Supplementary File S3.pdf]

Results of Next generation sequencing – Overview of the most dysregulated exomiRs

Name	Log2 fold change	Fold change	P-value	FDR p-value
hsa-miR-4516	2.301979591	4.93133954	2.60E-04	0.044631791
hsa-miR-16-5p	0.601698752	1.517502352	0.056039447	0.056039447
hsa-miR-143-3p	1.205078117	2.305497525	1.70E-03	0.097596967
hsa-miR-451a	1.234470352	2.352949491	4.46E-03	NaN
hsa-miR-151a-5p	-0.859726972	-1.814694849	5.46E-03	0.076437562
hsa-miR-223-3p	1.329213947	2.512657354	9.57E-04	0.099003501
hsa-miR-12135	-2.37776282	-5.19730174	1.36E-04	0.044631791
