# Supplementary material for: Liquid Biopsy Analysis of the EV-Associated Micro-RNA Signature in Vulvar Carcinoma May Benefit Disease Diagnosis and Prognosis
Source: Cancers (Basel). 2026 Jan 29;18(3):438. doi: 10.3390/cancers18030438 (PMC12896608; doi:10.3390/cancers18030438)
Supplement: Supplementary file 1 [file cancers-18-00438-s001.zip › Supplementary File S4.pdf]

Analysis of the correlation between exomiR expression and age of participants

1. Correlation between exomiR expression and age across all participants

The general correlation between exomiR expression levels and age across all participants - including vulvar cancer patients as well as healthy controls - was analyzed using Pearson's correlation coefficient. The results are shown in the respective tables for each of the detected target exomiR and for the stably expressed housekeeping gene.

A) miR-451a

		Age	log2-451a
Age	Pearson Correlation	1	.166*
	Sig. (2-tailed)		.050
	N	141	141
log2-451a	Pearson Correlation	.166*	1
	Sig. (2-tailed)	.050	
	N	141	141

*. Correlation is significant at the 0.05 level (2-tailed).

B) miR-16-5p

		Age	log2-16-5p
Age	Pearson Correlation	1	.062
	Sig. (2-tailed)		.463
	N	141	141
log2-16-5p	Pearson Correlation	.062	1
	Sig. (2-tailed)	.463	
	N	141	141

C) miR-151a-5p

		Age	log2-151a-5p
Age	Pearson Correlation	1	.042
	Sig. (2-tailed)		.625
	N	141	140
log2-151a-5p	Pearson Correlation	.042	1
	Sig. (2-tailed)	.625	
	N	140	140

D) miR-4516

		Age	log2-4516
Age	Pearson Correlation	1	-.059
	Sig. (2-tailed)		.489
	N	141	140
log2-4516	Pearson Correlation	-.059	1
	Sig. (2-tailed)	.489	
	N	140	140

E) miR-223-3p

		Age	log2-223-3p
Age	Pearson Correlation	1	.032
	Sig. (2-tailed)		.707
	N	141	141
log2-223-3p	Pearson Correlation	.032	1
	Sig. (2-tailed)	.707	
	N	141	141

F) miR-143-3p

		Age	log2-143-3p
Age	Pearson Correlation	1	-.012
	Sig. (2-tailed)		.888
	N	141	135
log2-143-3p	Pearson Correlation	-.012	1
	Sig. (2-tailed)	.888	
	N	135	135

G) miR-378a-3p (Endogenous control)

		Age	378a-3p
Age	Pearson Correlation	1	-.009
	Sig. (2-tailed)		.949
	N	60	60
378a-3p	Pearson Correlation	-.009	1
	Sig. (2-tailed)	.949	
	N	60	60

2. Correlation between exomiR expression and age in healthy controls
The correlation between exomiR expression levels and age was analyzed in exclusively the healthy control cohort, also using Pearson's correlation coefficient. The results are shown in the respective tables for each of the detected target exomiR and for the stably expressed housekeeping gene.

A) miR-451a

		Age	log2-451a
Age	Pearson Correlation	1	-.185
	Sig. (2-tailed)		.156
	N	60	60
log2-451a	Pearson Correlation	-.185	1
	Sig. (2-tailed)	.156	
	N	60	60

B) miR-16-5p

		Age	log2-16-5p
Age	Pearson Correlation	1	.012
	Sig. (2-tailed)		.930
	N	60	60
log2-16-5p	Pearson Correlation	.012	1
	Sig. (2-tailed)	.930	
	N	60	60

C) miR-151a-5p

		Age	log2-151a-5p
Age	Pearson Correlation	1	-.171
	Sig. (2-tailed)		.195
	N	60	59
log2-151a-5p	Pearson Correlation	-.171	1
	Sig. (2-tailed)	.195	
	N	59	59

D) miR-4516

		Age	log2-4516
Age	Pearson Correlation	1	-.101
	Sig. (2-tailed)		.447
	N	60	59
log2-4516	Pearson Correlation	-.101	1
	Sig. (2-tailed)	.447	
	N	59	59

E) miR-223-3p

		Age	log2-223-3p
Age	Pearson Correlation	1	.061
	Sig. (2-tailed)		.644
	N	60	60
log2-223-3p	Pearson Correlation	.061	1
	Sig. (2-tailed)	.644	
	N	60	60

F) miR-143-3p

		Age	log2-143-3p
Age	Pearson Correlation	1	-.137
	Sig. (2-tailed)		.309
	N	60	57
log2-143-3p	Pearson Correlation	-.137	1
	Sig. (2-tailed)	.309	
	N	57	57

G) miR-378a-3p (Endogenous control)

		Age	378a-3p
Age	Pearson Correlation	1	-.009
	Sig. (2-tailed)		.949
	N	60	60
378a-3p	Pearson Correlation	-.009	1
	Sig. (2-tailed)	.949	
	N	60	60

3. Correlation between exomiR expression and age in vulvar cancer patients
Correspondingly, the correlation between exomiR expression levels and age was analyzed in exclusively the vulvar cancer patient cohort, also using Pearson's correlation coefficient. The results are shown in the respective tables for each of the detected target exomiR and for the stably expressed housekeeping gene.

A) miR-451a

		Age	log2-451a
Age	Pearson Correlation	1	.169
	Sig. (2-tailed)		.132
	N	81	81
log2-451a	Pearson Correlation	.169	1
	Sig. (2-tailed)	.132	
	N	81	81

B) miR-16-5p

		Age	log2-16-5p
Age	Pearson Correlation	1	.049
	Sig. (2-tailed)		.662
	N	81	81
log2-16-5p	Pearson Correlation	.049	1
	Sig. (2-tailed)	.662	
	N	81	81

C) miR-151a-5p

		Age	log2-151a-5p
Age	Pearson Correlation	1	.011
	Sig. (2-tailed)		.922
	N	81	81
log2-151a-5p	Pearson Correlation	.011	1
	Sig. (2-tailed)	.922	
	N	81	81

D) miR-4516

		Age	log2-4516
Age	Pearson Correlation	1	.079
	Sig. (2-tailed)		.485
	N	81	81
log2-4516	Pearson Correlation	.079	1
	Sig. (2-tailed)	.485	
	N	81	81

E) miR-223-3p

		Age	log2-223-3p
Age	Pearson Correlation	1	-.141
	Sig. (2-tailed)		.210
	N	81	81
log2-223-3p	Pearson Correlation	-.141	1
	Sig. (2-tailed)	.210	
	N	81	81

F) miR-143-3p

		Age	log2-143-3p
Age	Pearson Correlation	1	-.102
	Sig. (2-tailed)		.373
	N	81	78
log2-143-3p	Pearson Correlation	-.102	1
	Sig. (2-tailed)	.373	
	N	78	78

G) miR-378a-3p (Endogenous control)

		Age	378a-3p
Age	Pearson Correlation	1	.080
	Sig. (2-tailed)		.475
	N	81	81
378a-3p	Pearson Correlation	.080	1
	Sig. (2-tailed)	.475	
	N	81	81
