# Supplementary material for: Liquid Biopsy Analysis of the EV-Associated Micro-RNA Signature in Vulvar Carcinoma May Benefit Disease Diagnosis and Prognosis
Source: Cancers (Basel). 2026 Jan 29;18(3):438. doi: 10.3390/cancers18030438 (PMC12896608; doi:10.3390/cancers18030438)
Supplement: Supplementary file 1 [file cancers-18-00438-s001.zip › Supplementary File S5.pdf]

Survival Table - Kaplan-Meier analysis by nodal status

Nodal status		Time	Status	Cumulative Proportion Surviving at the Time		N of Cumulative Events	N of Remaining Cases
				Estimate	Std. Error		
negative	1	5.000	0	.	.	0	38
	2	5.000	0	.	.	0	37
	3	5.000	0	.	.	0	36
	4	6.000	0	.	.	0	35
	5	6.000	0	.	.	0	34
	6	7.000	0	.	.	0	33
	7	7.000	0	.	.	0	32
	8	7.000	0	.	.	0	31
	9	7.000	0	.	.	0	30
	10	7.000	0	.	.	0	29
	11	19.000	0	.	.	0	28
	12	49.000	0	.	.	0	27
	13	67.000	1	.963	.036	1	26
	14	105.000	0	.	.	1	25
	15	130.000	0	.	.	1	24
	16	205.000	1	.923	.052	2	23
	17	234.000	0	.	.	2	22
	18	241.000	0	.	.	2	21
	19	243.000	0	.	.	2	20
	20	286.000	0	.	.	2	19
	21	393.000	0	.	.	2	18
	22	411.000	0	.	.	2	17
	23	438.000	0	.	.	2	16
	24	474.000	0	.	.	2	15
	25	535.000	0	.	.	2	14
	26	579.000	0	.	.	2	13
	27	639.000	1	.852	.084	3	12
	28	642.000	1	.781	.102	4	11
	29	734.000	0	.	.	4	10
	30	822.000	0	.	.	4	9
	31	861.000	0	.	.	4	8
	32	871.000	0	.	.	4	7
	33	1,022.000	0	.	.	4	6
	34	1,068.000	0	.	.	4	5
	35	1,263.000	0	.	.	4	4
	36	1,478.000	0	.	.	4	3
	37	1,576.000	0	.	.	4	2
	38	2,435.000	0	.	.	4	1
	39	2,442.000	0	.	.	4	0
positive	1	7.000	0	.	.	0	34
	2	11.000	0	.	.	0	33
	3	20.000	0	.	.	0	32
	4	40.000	1	.969	.031	1	31
	5	51.000	1	.938	.043	2	30
	6	80.000	0	.	.	2	29
	7	90.000	0	.	.	2	28
	8	99.000	1	.904	.053	3	27
	9	125.000	1	.871	.061	4	26
	10	126.000	0	.	.	4	25
	11	151.000	0	.	.	4	24
	12	157.000	1	.834	.068	5	23
	13	179.000	1	.798	.074	6	22
	14	210.000	0	.	.	6	21
	15	221.000	1	.760	.080	7	20
	16	224.000	0	.	.	7	19
	17	273.000	1	.720	.085	8	18
	18	314.000	0	.	.	8	17
	19	350.000	1	.678	.090	9	16
	20	350.000	0	.	.	9	15
	21	381.000	1	.632	.095	10	14
	22	417.000	1	.587	.098	11	13
	23	526.000	0	.	.	11	12
	24	561.000	1	.538	.101	12	11
	25	610.000	1	.489	.103	13	10
	26	622.000	0	.	.	13	9
	27	636.000	0	.	.	13	8
	28	956.000	1	.428	.107	14	7
	29	1,168.000	0	.	.	14	6
	30	1,211.000	1	.357	.110	15	5
	31	1,223.000	1	.285	.109	16	4
	32	1,361.000	1	.214	.102	17	3
	33	1,493.000	0	.	.	17	2
	34	1,644.000	0	.	.	17	1
	35	2,054.000	0	.	.	17	0
